# Comparative Transcriptome Analysis of a Toxin-Producing Dinoflagellate *Alexandrium* *catenella* and Its Non-Toxic Mutant

**DOI:** 10.3390/md12115698

**Published:** 2014-11-24

**Authors:** Yong Zhang, Shu-Fei Zhang, Lin Lin, Da-Zhi Wang

**Affiliations:** State Key Laboratory of Marine Environmental Science, College of the Environment and Ecology, Xiamen University, Xiamen 361102, China; E-Mails: foryzhy@gmail.com (Y.Z.); shufeizhang@qq.com (S.-F.Z.); linlin1982@xmu.edu.cn (L.L.)

**Keywords:** marine dinoflagellates, *Alexandrium catenella*, paralytic shellfish toxins, mutant, toxin biosynthesis, transcriptome, RNA-seq

## Abstract

The dinoflagellates and cyanobacteria are two major kingdoms of life producing paralytic shellfish toxins (PSTs), a large group of neurotoxic alkaloids causing paralytic shellfish poisonings around the world. In contrast to the well elucidated PST biosynthetic genes in cyanobacteria, little is known about the dinoflagellates. This study compared transcriptome profiles of a toxin-producing dinoflagellate, *Alexandrium catenella* (ACHK-T), and its non-toxic mutant form (ACHK-NT) using RNA-seq. All clean reads were assembled *de novo* into a total of 113,674 unigenes, and 66,812 unigenes were annotated in the known databases. Out of them, 35 genes were found to express differentially between the two strains. The up-regulated genes in ACHK-NT were involved in photosynthesis, carbon fixation and amino acid metabolism processes, indicating that more carbon and energy were utilized for cell growth. Among the down-regulated genes, expression of a unigene assigned to the long isoform of *sxtA*, the initiator of toxin biosynthesis in cyanobacteria, was significantly depressed, suggesting that this long transcript of *sxtA* might be directly involved in toxin biosynthesis and its depression resulted in the loss of the ability to synthesize PSTs in ACHK-NT. In addition, 101 putative homologs of 12 cyanobacterial *sxt* genes were identified, and the *sxtO* and *sxtZ* genes were identified in dinoflagellates for the first time. The findings of this study should shed light on the biosynthesis of PSTs in the dinoflagellates.

## 1. Introduction

Paralytic shellfish toxins (PSTs) are a group of neurotoxic alkaloids, including saxitoxin (STX) and its 57 analogs, specifically blocking the voltage-gated sodium channel and resulting in paralytic shellfish poisonings (PSPs) around the world with an estimated 2000 annual toxic cases in humans globally [1]. With the recent increase of PST-producing algal blooms in terms of their frequency, intensity and geographic distribution, PSPs are now considered to be a serious toxicological health risk affecting humans, other animals, and ecosystems [2].

PSTs are produced by two kingdoms of life, eukaryotic marine dinoflagellates and prokaryotic freshwater cyanobacteria. Several dinoflagellate species belonging to the genera *Alexandrium*, *Pyrodinium* and *Gymnodinium* are able to produce PSTs [3,4] and, in freshwater environments, several filamentous species of cyanobacteria from the genera *Anabaena*, *Aphanizomenon*, *Lyngbya* and *Cylindrospermopsis* are known to produce PSTs [5,6,7,8]. Much effort has been devoted to investigating the mechanisms of STX biosynthesis in both dinoflagellates and cyanobacteria and, recently, a gene cluster responsible for toxin biosynthesis in the cyanobacterium *Cylindrospermopsis raciborskii* T3 was unveiled [9]. With a length more than 35 kb, cluster *sxt* contains all of the 26 genes potentially participating in STX biosynthesis and, based on open reading frames (ORFs) of *sxt* and biosynthetic intermediates, a bird’s-eye view of the toxin biosynthesis mechanism is presented and a revised pathway is proposed based on a previous hypothetical procedure of STX synthesis [9]. To date, the *sxt* gene cluster is also identified in several other cyanobacterial species [10,11,12].

Nonetheless, STX biosynthesis in dinoflagellates is still elusive. The STX biosynthesis mechanism of dinoflagellates is considered to be similar to cyanobacteria on account of the identical precursor incorporation patterns and stereochemistry [13,14,15]. Precursors for STX biosynthesis are arginine, *S*-adenosylmethionine (SAM) and acetate for both organisms [13,16]. However, identifying the genes involved in STX biosynthesis in the dinoflagellates is not as effortless as in cyanobacteria owing to the enormous genome and high copy number of genes in dinoflagellates [17,18]. In a previous study with SAM synthetase targeted, the degenerate PCR method was utilized to amplify possible STX-related genes, but it failed [19]. An expressed sequence tag library was constructed and toxic strains were compared with non-toxic strains of *Alexandrium minutum* using microarray, but the differential expression genes are not directly related to toxin biosynthesis [20]. Since the recent application of high-throughput sequencing, homologs of cyanobacterial *sxt* genes are found to be transcribed in two toxin-producing *Alexandrium* strains [21]. Based on these homologs, *sxtA* and *sxtG* which putatively participate in the first two steps of STX biosynthesis are amplified and characterized [21,22]. The *sxtA* in dinoflagellates is transcribed into two types of transcripts: a long transcript contains *sxtA1*-*sxtA4* domains, the same as that in cyanobacteria, while a short transcript possesses simply *sxtA1*-*sxtA3* domains. Comparison between toxic and non-toxic dinoflagellate species indicates that *sxtA1* and *sxtA4* may participate in toxin biosynthesis [21]. Phylogenetic analysis suggests that the C-terminal of sxtA and sxtG are critical for STX synthesis, but it is not conclusive as to whether they are specific to toxin-producing dinoflagellates [23]. Toxin biosynthetic genes have also been looked for in STX producing strains and their non-toxic mutants in several studies. With identical genetic profiles, mutants and their original wild strains are expected to be ideal materials for identifying toxin biosynthesis genes [24]. Mutant genes are considered to be involved in STX synthesis, but differentially expressed genes (DEGs) identified by subtractive hybridization between toxic and non-toxic subclones from a parental toxin-producing *A. tamarense* present no direct relationship with loss of toxicity [24]. In addition to studies of toxin related genes, some efforts have been devoted to mining proteins potentially participating in STX biosynthesis, and several candidate proteins are identified from *A. catenella* [25,26]. However, relationships between these proteins and STX genes remain to be ascertained [27].

In our study, next generation sequencing (NGS), RNA-seq, was applied to compare wild type toxin-producing *A. catenella* (ACHK-T) and its non-toxin producing mutant (ACHK-NT). The purpose of this study was to reveal possible causes of the disability to synthesize STX in the mutant strain and the potential genes involved in toxin biosynthesis in marine dinoflagellates.

**Figure 1:**
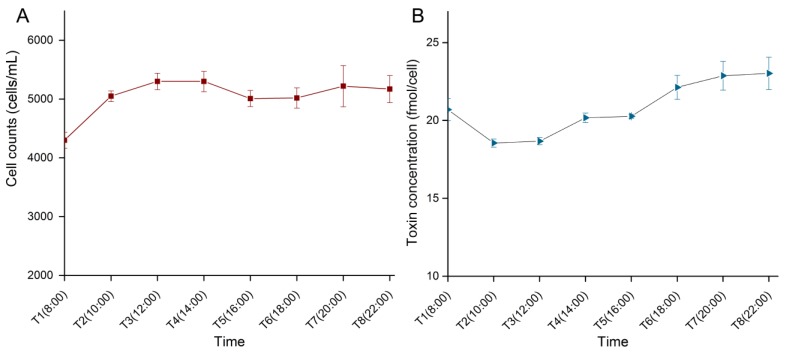
Cell density and toxin content of *Alexandrium catenella* (ACHK-T) during the light period. (**A**) Cell density; (**B**) Toxin content. Variation of toxin content indicated that saxitoxin (STX) was synthesized during the light period.

## 2. Results

### 2.1. Cell Density and Toxin Content during the Light Period

Variations of cell density and toxin content of ACHK-T during the light period are shown in Figure 1. Cell density increased from 08:00 to 10:00, and then maintained a stable level. While toxin content decreased from 08:00 to 10:00 and increased gradually during the rest light period, suggesting that toxin biosynthesis proceeded in this period. Based on these results combined with cell cycle analysis of ACHK-T and ACHK-NT (Supplementary Figure S2), cells at the same cell cycle phase (time point T5, 16:00) were collected for RNA extraction and RNA-seq analysis.

### 2.2. RNA-seq and de novo Assembly

Sequenced on an Illumina Hiseq^TM^ 2000 (Illumina, San Diego, CA, USA), a total of 130,894,006 and 128,609,864 paired-end raw reads with a read length of 90 bp were generated for ACHK-NT and ACHK-T, respectively. Reads containing adapter, poly-N and low quality reads were removed before data analysis. The Q20 of the clean reads data ranged from 93.75% to 96.88% and GC content of either left end or right end reads was constant at about 61% for the two samples. Using these clean reads, *de novo* transcriptome assembly was performed using Trinity with min_kmer_cov set to 2 and other parameters set to default [28]. A total of 155,353 transcripts were obtained, which had a mean length of 1003 bp and N50 was 1549 bp in length. 61,115 transcripts were more than 1000 bp in length and 18,233 transcripts were more than 2000 bp. All transcripts were clustered into 113,674 unique transcripts (unigenes) and the average length of the unigenes was 901 bp, with an N50 length of 1462 bp. 41,614 unigenes were more than 1000 bp (Table 1). 99,298 and 101,105 unigenes were obtained from ACHK-T and ACHK-NT, respectively. The assembly results and uniform distribution examination (Supplementary FigureS3) indicated a comparable quality of RNA-seq data as reported [29].

**Table 1:** Summary of *A. catenella* transcriptome.

Items	Value
Total number of raw reads	259,503,870
Total number of clean reads	242,852,652
Total clean nucleotides (Gb)	24.28
Q20 percentage (%)	93.75~96.88
GC percentage (%)	61.07~61.40
Total number of transcripts	155,353
Mean length of transcripts	1003
N50 length of transcripts	1549
Total number of unigenes	113,674
Mean length of unigenes	901
N50 length of unigenes	1462

### 2.3. Gene Functional Annotation

To predict the functions of unigenes, BLASTX was performed against databases including: the NCBI non-redundant protein sequences (NR) database, eukaryotic clusters of Orthologous Groups (KOG), Kyoto Encyclopedia of Genes and Genomes (KEGG) and the Swiss-Prot database. BLASTN was carried out against the NCBI non-redundant nucleotide sequences (NT) database. With a threshold e-value ≤ 1e^−5^, 40,535 of the 113,674 unigenes (35.65%) had significant matches in the KOG database and a total of 66,812 unigenes (58.77%) were successfully annotated at least in one database (Table 2).

The 40,535 unigenes annotated in KOG were classified into 26 categories including an unnamed protein group (Supplementary Figure S4). Among these groups, signal transduction was the largest group and contained 7260 unigenes, followed by the extracellular structure and the general functional prediction only group. RNA processing and modification, cytoskeleton and post-translational modification, protein turnover, and chaperon covered a majority of unigenes as well, each of them possessing more than 3500 unigenes. Only 39 unigenes were categorized into cell motility, as well as an unnamed protein group.

To classify the function of unigenes, gene ontology (GO) annotation was performed with BLAST2GO. Out of 34,372 unigenes matched in the NR database, 28,146 genes obtained GO terms and were subsequently classified into three functional categories (Supplementary Figure S5). Among the categories of biological processes, the cellular process and metabolic process were the two dominant groups. For the cellular component, the top GO terms were cell and cell part, followed by organelle. The most represented groups in the molecular function term were binding and catalytic activity.

**Table 2:** Annotation results of unigenes against different databases.

*Database*	Number of Unigenes
NR	34,372
NT	2071
KEGG	10,362
SwissProt	23,511
Pfam	52,026
GO	28,146
KOG	40,535
At least in one database	66,812

Annotated GO terms were then applied to assign unigenes associated EC (Enzyme Commission) numbers. A total of 5195 transcripts were assigned to 924 EC numbers involved in 194 pathways. The top ten represented pathways are shown in Supplementary Figure S6A, and the most represented pathway was protein processing in the endoplasmic reticulum which covered 63 enzymes matched by 358 unigenes, followed by ribosome and purine metabolism, with 309 and 255 unigenes participating in the pathways across 101 and 94 enzymes, respectively. In addition, the KEGG Automatic Annotation Server (KAAS) annotation provided 10,362 unigenes with 1738 unique KEGG Orthology (KO) codes, and kinase, transferase, calmodulin, enolase, heat shock protein (HSP) and dehydrogenase dominated the top 10 KOs (Supplementary Figure S6B). Based on the secondary pathway hierarchy, all the transcripts were classified into 22 groups, and abundant genes were involved in signal transduction as is known in dinoflagellates (Supplementary Figure S7) [14].

In order to elucidate potential gene functions, the ORF of transcripts was predicted and the coding region sequences were then translated into amino acid sequences, followed by searching against the Pfam (Protein family) database. Functional domain and Pfam annotation were assigned to 52,026 unigenes within 4352 protein categories. Distribution of these unigenes *versus* different Pfam families is shown in Supplementary Figure S8A. Most families were found to possess a small number of unigenes. The top ten categories are listed in Supplementary Figure S8B. The dominant groups represented were the protein kinase domain and protein tyrosine kinase, and both of them participated in multiple cellular processes. Ankyrin repeat, together with tetratricopeptide repeat, commonly mediates interactions between proteins. Except for gammaherpesvirus capsid protein with unknown function, the rest of the protein families played various roles in a wide range of organisms.

### 2.4. Differential Expression Gene Analysis

**Figure 2:**
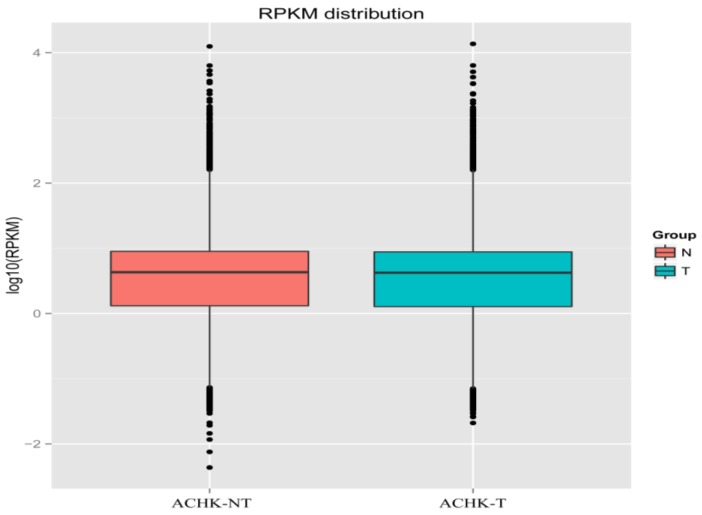
Boxplot of the log Reads Per Kilo bases per Million reads (RPKM) expression values in ACHK-NT and ACHK-T. Expression values between the two strains suggested a highly similar gene expression pattern.

To avoid the influence of gene length and sequencing depth on the accuracy of read count, the RPKM (Reads Per Kilo bases per Million reads) method was applied to carry out quantification of gene expression. Using RSEM, the read count value of each gene was obtained and then transformed into the RPKM [30]. Density distributions of the RPKM from the two samples were compared to examine the expression pattern of genes using a boxplot with the log RPKM values. The median and quartile values of RPKM between the samples were practically identical (Figure 2), indicating a highly similar expression pattern of ACHK-T and ACHK-NT. A total of 96,735 co-expression unigenes were detected in the two samples with a threshold of RPKM > 0.3 and length > 200 bp. Although specifically expressed genes were obtained in each sample, most of these genes were proved to be false positives between ACHK-T and ACHK-NT under the strict threshold. Differential expression analysis of genes was performed on DEGSeq with data of read counts normalized using the Trimmed Mean of M-values method [31]. Using the cut-off described in “Experimental Section 4.5”, 13 unigenes were down-regulated and 22 unigenes were up-regulated in ACHK-NT compared to ACHK-T (Table 3, Supplementary Table S1).

**Table 3:** Differentially expressed genes between ACHK-NT and ACHK-T, including normalized read count of unigenes, expression fold change and non-redundant (NR) annotation. More information and annotations against other databases are shown in Supplementary Table S1.

Gene_ID	N_Readcount	T_Readcount	log2.Fold_Change	NR Description
*Down-regulation in ACHK-NT:*				
comp66063_c0	2	37391.29	−14.226	exoglucanase 3 1,4-beta-cellobiohydrolase3 family GH6, Exoglucanase 3 (*Pyrenophora tritici-repentis* (strain Pt-1C-BFP)), Predicted CDS Pa_4_2420 (*Podospora anserina*) [*Chondrus crispus*]
comp66169_c0	1	12503.81	−13.646	SxtA long isoform precursor [*Alexandrium fundyense*]
comp57121_c1	3	15706	−12.39	putative uncharacterized protein [*Sutterella wadsworthensis* CAG:135]
comp66059_c0	3	3644.78	−10.282	hypothetical protein Pmar_PMAR010035 [*Perkinsus marinus* ATCC 50983]
comp47304_c0	15	4102.99	−8.131	cell wall-associated hydrolase [*Burkholderia multivorans* ATCC 17616]
comp57121_c0	0	4113	−7.3658	hypothetical protein Csp_D29540 [Curvibacter putative symbiont of *Hydra magnipapillata*]
comp62287_c0	28.75	3542.4	−6.9805	hypothetical protein, partial [*Escherichia coli*]
comp64637_c0	0	2734.05	−6.7766	enzymatic polyprotein; Endonuclease; Reverse transcriptase, putative [*Pediculus humanus corporis*]
comp46928_c0	15	1300.95	−6.4739	Protein CBR-LARP-1 [*Caenorhabditis briggsae]*
comp114480_c0	180	1354	−2.9466	putative S12 family peptidase [*Gemmatimonas aurantiaca* T-27]
comp66521_c0	11289.22	28698.06	−1.3815	Ankyrin repeats containing protein [delta proteobacterium BABL1]
comp60385_c0	2671.63	5630.9	−1.1111	--
comp47283_c1	15773.39	25201.15	−0.71144	RecName: Full = Photosystem I P700 chlorophyll a apoprotein A2; AltName: Full = PSI-B; AltName: Full = PsaB
*Up-regulation in ACHK-NT:*				
comp64975_c0	119074.04	74056.39	0.64972	cytochrome c oxidase subunit 1 [*Alexandrium catenella*]
comp65226_c1	19727.54	12087.19	0.67129	light-harvesting protein, partial [*Symbiodinium* sp. clade C3]
comp54599_c0	40289.85	24201.67	0.69987	--
comp65353_c0	11048.61	6220.45	0.79333	hypothetical protein NCLIV_047860 [*Neospora caninum* Liverpool]
comp63840_c0	8189.47	4453.25	0.84347	probable high CO2 inducible periplasmic protein [*Heterocapsa triquetra*]
comp65627_c0	31244.01	16909.13	0.85033	proliferating cell nuclear antigen [*Alexandrium affine*]
comp66053_c0	60305.52	30993.93	0.92486	plastid C1 class II fructose bisphosphate aldolase [*Heterocapsa triquetra*]
comp66077_c1	8811.01	4410	0.96308	hypothetical protein RFI_22066 [*Reticulomyxa filosa*]
comp65597_c0	8402.67	4024.29	1.0267	hypothetical protein [*Verrucomicrobiae* bacterium DG1235]
comp65950_c0	5831.47	2682.38	1.0849	PREDICTED: phosphoserine aminotransferase-like [*Anolis carolinensis*]
comp33299_c0	5150	2360	1.0903	Eukaryotic translation initiation factor 3 subunit 9, putative [*Eimeria acervulina*]
comp70851_c0	2940.5	975	1.5571	Calcium-dependent protein kinase, putative [*Perkinsus marinus* ATCC 50983]
comp71917_c0	2656.96	861	1.5902	--
comp62882_c0	14038.72	4456	1.6201	SCO-spondin [*Crassostrea gigas*]
comp63141_c0	11545.27	3106	1.8587	hypothetical protein Rcas_3705 [*Roseiflexus castenholzii* DSM 13941]
comp65143_c0	58658.95	11917.14	2.2639	light-harvesting protein, partial [*Symbiodinium* sp. clade C3]
comp65099_c0	3250.62	428.7	2.8872	Phosphoserine aminotransferase, putative [*Perkinsus marinus* ATCC 50983]
comp47128_c0	956.92	52	4.1664	--
comp64715_c0	1765.01	55	4.9687	--
comp57722_c1	872	0	5.0926	--
comp57938_c1	1421.88	19	6.1902	*S*-adenosyl-homocysteine hydrolase like protein, partial [*Alexandrium fundyense*]
comp64901_c0	1290	10	6.9758	--

The up-regulated genes were mainly involved in photosynthesis, carbon fixation, amino acid metabolism, oxidative phosphorylation and translation processes, including: light-harvesting proteins, fructose bisphosphate aldolase, phosphoserine aminotransferase, cytochrome c oxidase and translation initiation factor 3. The expressions of a proliferating cell nuclear antigen gene and a high CO_2_ inducible periplasmic protein gene were also enhanced. The calcium-dependent protein kinase (CDPK) gene was also up-regulated in ACHK-NT, which plays fundamental roles in signaling and regulatory processes. In addition, the expression of an *S*-adenosyl-homocysteine hydrolase (SAHH) like protein gene was significantly enhanced in ACHK-NT.

The majority of the down-regulated genes were classified as hypothetical function or function unknown. In ACHK-NT, unigenes assigned to 1,4-beta-cellobiohydrolase, *sxtA* long isoform precursor and cell wall-associated hydrolase were down-regulated. 1,4-beta-cellobiohydrolase generally functions in carbohydrate metabolic processes, while the exact function of cell wall-associated hydrolase is difficult to confirm since it comprises various enzymes with diverse substrate specificities [32]. The *sxtA* long isoform is the starting gene of the STX biosynthesis pathway, down-regulation of which implied its relationship with the loss of ability to produce STX in ACHK-NT.

### 2.5. Identification of STX Genes

**Figure 3:**
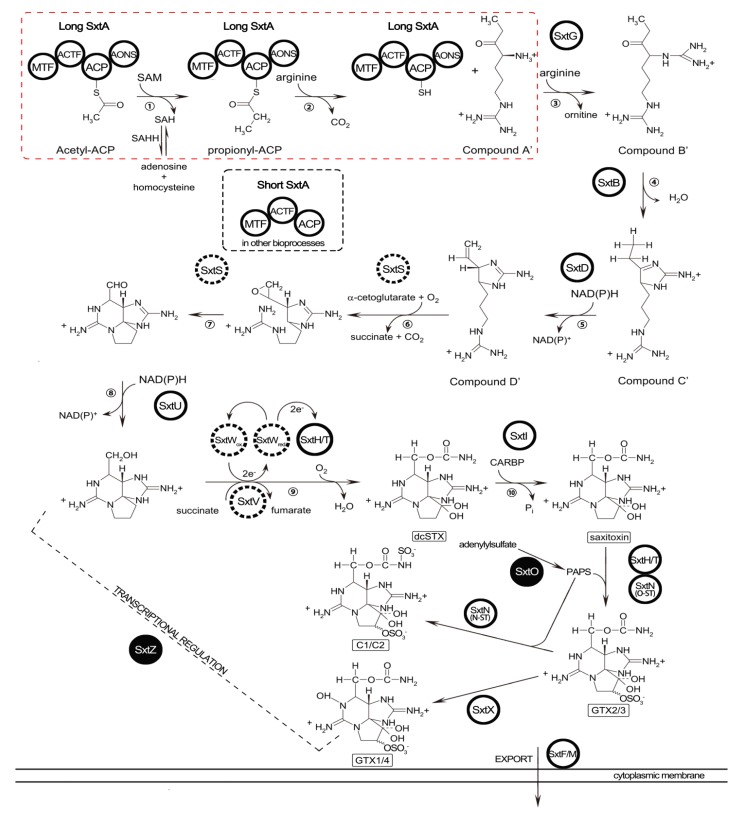
Revised pathway of saxitoxin biosynthesis in *A. catenella* (Cited and modified from previous reports [10,27]). The *sxt* genes identified are in circles and those not identified in this study are in dashed circles. *sxt* genes identified for the first time in dinoflagellates are labeled in solid circles: *sxtO* was involved in conversion of STX analogs; *sxtZ* participated in regulation of STX biosynthesis. STX biosynthesis steps in a red dashed box represent terminated reactions due to depressed expression of *sxtA* long transcripts in ACHK-NT, which resulted in the ability loss of toxin production.

The searching strategy applied to identify genes potentially involved in STX biosynthesis is described in “Experimental Section 4.6”. BLASTN searching against our transcriptome dataset provided no homologous sequences to the *sxt* genes even though an e-value < 0.1 was applied. To inspect this BLASTN result, sequence alignments were attempted subsequently, and both the *sxtA* and *sxtG* genes identified in *Alexandrium* spp. were successfully annotated. Unigene comp66169_c0 was aligned with *sxtA* sequences of the cyanobacteria or dinoflagellates. Similar alignment was also carried out for the unigene comp63408_c0 which was assigned to the *sxtG* gene. These two unigenes exhibited high similarity with those of *Alexandrium* spp.; however, neither had significant hits when compared with cyanobacterial *sxtA* and *sxtG*. Nonetheless, TBLASTN searches presented better results and totally 101 homologs of 12 *sxt* proteins from cyanobacteria were obtained (Table 4, and Supplementary Table S2). Except for *sxtS*, seven other genes (*sxtA*, *sxtB*, *sxtD*, *sxtG*, *sxtH/T*, *sxtI* and *sxtU*) directly involved in the STX pathway were putatively identified. Out of the other six core toxin genes (*sxtC*, *sxtE*, *sxtF*, *sxtP*, *sxtQ* and *sxtR*) in the cyanobacteria [1,33], only *sxtF* (/*sxtM*) was found. Furthermore, several putative homologs of *sxtO* and *sxtZ* related to the conversion of STX analogs and transcriptional regulation of STX production, respectively, were also identified, which were not previously found in dinoflagellates [9,14,23]. All of these putative toxin related genes are presented in a revised STX biosynthesis pathway based on the previously proposed pathway (Figure 3) [10].

2.6. qRT-PCR Validation of DEGs

**Figure 4:**
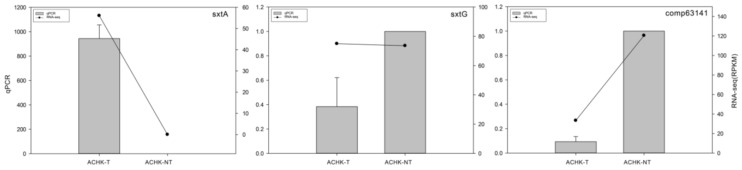
Quantitative RT-PCR validations of differentially expressed genes. Gene expression of ACHK-T was normalized to that of ACHK-NT where the expression value was set as 1. The housekeeping gene *calm* was chosen as internal reference. The error bar represented the standard deviation of biological replicates.

Quantitative RT-PCR (qRT-PCR) was performed to examine the transcription levels of DEG outcomes from RNA-seq. The housekeeping gene calmodulin (*calm*, NCBI accession number: EF133873) was chosen as the reference gene to normalize expression levels between the two strains. The characterized toxin-related genes *sxtA*, *sxtG* and an up-regulated gene comp63141 were selected to compare, and the *sxtA* and comp63141 presented similar expression patterns with the RNA-seq results. However, the expression of *sxtG* showed some inconsistency between qRT-PCR and RNA-seq results (Figure 4).

**Table 4:** BLAST analysis of potential STX genes in *A. catenella*. Numbers represented homologs of cyanobacterial *sxt* genes identified by TBLASTN search.

STX Genes	Putative Function	*A. catenella* Unigenes
*sxtA*	Aspartate aminotransferase	9
*sxtB*	Cytidine deaminase	2
*sxtD*	Sterole desaturase	1
*sxtF/M*	Toxic compound efflux protein	2
*sxtG*	Amidinotransferase	2
*sxtH/T*	Phenylpropionate dioxygenase	15
*sxtI*	O-carbamoyltransferase	2
*sxtN*	Sulfotransferase	1
*sxtO*	Adenylylsulfate kinase	1
*sxtU*	Short-chain alcohol dehydrogenase	59
*sxtX*	Cephalosporin hydroxylase	1
*sxtZ*	Two-component sensor histidine kinase	6

## 3. Discussion

In recent years, NGS technology has been considered to be a more effective approach for transcriptome studies. New *de novo* transcriptome assembly programs have been developed to adapt this technology and provide a powerful tool for genomic studies of species without genome information [28,34]. This study represented the first comparison of a toxigenic *A. catenella* and its non-toxic mutant using NGS technology and the *de novo* assembly method. A total number of 113,674 unigenes were obtained, with a comparable number of assembled unigenes as previously reported [29]. These unigenes might represent a majority of the genes existing in *A. catenella*, based on the prediction of approximately 90,000 genes in the largest dinoflagellate genomes [35]. Of the 113,674 transcripts, 58.77% was annotated at least in one database employed here. Nonetheless, as other studies mention [36], a portion of the functional annotated unigenes was assigned to be hypothetical or predicted proteins when BLAST against NR. Despite the highly similar gene expression patterns between ACHK-T and ACHK-NT, a total of 35 genes were found to express differentially.

### 3.1. Up-Regulated Genes in ACHK-NT

In ACHK-NT, genes involved in cellular metabolism, and encoding, for example fructose bisphosphate aldolase, light-harvesting protein and cytochrome c oxidase subunit І, exhibited higher expressions than in ACHK-T. Fructose bisphosphate aldolase is implicated in carbon fixation of photosynthesis and up-regulation of this gene indicated a more active carbon fixation and higher requirement for ATP in the mutant ACHK-NT. As a light receptor, the higher expression of light-harvesting protein captured and delivered more excitation energy to the photosystems and thus provided more energy for the synthesis of the ATP or NADPH required for carbon fixation. In addition, the cytochrome c oxidase subunit І gene participating in oxidative phosphorylation of cellular respiration was up-regulated in ACHK-NT. This enzyme plays a key role in aerobic metabolism and represents the terminal, energy-transfer proteins of respiration chains. Together with up-regulation of a probable high CO_2_ inducible periplasmic protein, an enhancement of respiration might occur in ACHK-NT. Similar variations of these cellular metabolisms are also found at protein level in ACHK-T and its mutant [26]. In congruence with the effects of up-regulated proteins as reported, enhanced expression of genes probably contributed also to the high growth capacity of ACHK-NT through modifications at transcriptional level. Moreover, the expression of the CDPK gene increased in the mutant. CDPK plays a central role in cellular signal transductions responding to environmental changes and thus might function similarly to HSPs, which possibly protected cells from the potential stresses of a broken intracellular balance resulting from the loss of toxicity in the mutant as reported [26,37]. The phosphoserine aminotransferase gene was also up-regulated in the mutant and the production of P-serine by this enzyme is involved in serine biosynthesis [38]. Increased concentration of serine could be transformed into other amino acids such as glycine and cysteine or numerous intermediates utilized in various metabolic pathways.

SAHH, encoded by *Sahh*, is an enzyme participating in the activated methyl cycle and may function in the regulation of the SAM pathway or the intracellular concentration of *S*-adenosyl-homocysteine (SAH) [39]. This enzyme catalyzes the reversible hydration of SAH into adenosine and homocysteine. *Sahh* is considered to be indirectly related to STX biosynthesis, whereas the exact property of the relationship is ambiguous [14,39]. In the first step of the STX biosynthesis pathway, *sxtA1* catalyses the transfer of a methyl from the donor SAM and produces SAH (Figure 3) [9]. However, this reaction was probably terminated due to the depressed expression of the *sxtA* long transcript. Nonetheless, the *Sahh* gene was significantly up-regulated, suggesting that more SAH was then synthesized from adenosine and homocysteine through SAHH, instead of the terminated reaction being catalyzed by *sxtA1*. Therefore, we speculated that the first step of the STX pathway functions as not only the initiation of toxin biosynthesis, but also as the potential provider of SAH used for other biological processes.

### 3.2. Down-Regulated Genes in ACHK-NT

Cellulases hydrolyze the glycosidic bond between two or more carbohydrates and degrade cellulose which is contained in the plant cell wall as well as the dinoflagellate thecal plate [40,41,42]. Cellulases are found in some dinoflagellate species and they are hypothesized to be required for cell cycle progression or cell partitioning during mitosis [43,44]. A previous study reports that the hydrolyzed algal biomass can be utilized as sources of carbon or energy for growth of the algae themselves [45]. Recently, in a photosynthetic green microalga, cells are found to have the same ability as heterotrophs to secrete cellulases for hydrolysis of cellulosic materials into cellobiose and are then used as an external carbon source [46]. In our study, a gene encoding the major type of cellulases, 1,4-beta-cellobiohydrolase, was identified in both libraries. Since sampling here was at T5 (16:00) when most cells were in the G1 phase without division, rather than relating to cell partitioning, cellobiohydrolase was more likely to function as in microalgae, digesting cellulose contained in the thecal plates to provide additional carbon or energy for the *A. catenella* cells. Significant depression of this gene indicated that the manner of external carbon or energy utilization might no longer be necessary since metabolic activities such as photosynthesis have been enhanced, providing ACHK-NT cells with enough energy. However, further investigation should be devoted to confirming that this type of mechanism also exists in dinoflagellates.

Searches for genes involved in STX biosynthesis have been conducted in several *Alexandrium* species using various methods [19,20,21,24,39]. Despite these attempts, homologous genes of the *sxt* cluster were identified in *A. fundyense* and *A. minutum* only recently [21,23], based on the discovery of *sxt* gene clusters in the cyanobacteria [9,10,11]. As the starting gene for toxin biosynthesis, *sxtA* possesses two different transcripts in dinoflagellates. These two transcripts differ in length and the longer one contains all four domains as in cyanobacteria, while the shorter one has only three, without the last aminotransferase domain [21]. The exact functions of these two sorts of transcripts in STX biosynthesis are still vague. Based on the role that *sxtA4* plays in cyanobacteria, the longer transcript might more likely participate directly in toxin biosynthesis [21,36]. In our study, both isoforms of transcripts were obtained from the assembled unigenes. Both the long and short *sxtA* transcripts found shared sequence coverage of 99% with the reported long and short ones from *A. fundyense*. Alignment of both isoforms showed a similarity of 90.41% and 89.03% to the reported sequences, as well as similar GC contents (Supplementary Figure S9) [21].

Among the down-regulated genes, unigene comp66169_c0 was annotated as the long isoform of the dinoflagellate *sxtA* gene. The RPKM value of comp66169_c0 was 56.1 among the top 1% expressed genes, indicating a high expression level of *sxtA* in ACHK-T. However, only one read could be mapped back to comp66169_c0 for ACHK-NT. This single read might be either derived from genes which possess sequences of partial similarity with comp66169_c0, or as “background” under the threshold of RPKM < 0.3. Hence the long transcript of *sxtA*, comp66169_c0, was probably not transcribed in the non-toxic mutant. Besides the long *sxtA* transcript, unigene comp20666_c0 was assigned to the short isoform of *sxtA* in our study. The expression level of this short transcript presented no difference between ACHK-T and ACHK-NT, and the RPKM of the short transcript was much lower compared to the long transcript. Consequently, we proposed that the long *sxtA* transcript might directly participate in STX biosynthesis, while the short transcript potentially functioned in other biological processes. As the starting gene of toxin biosynthesis, down-regulation of the long transcript resulted in the breakdown of initial reactions in STX production and ultimately the loss of the ability to synthesize the toxin in ACHK-NT, as shown in the red dashed box in Figure 3. 

A recent study reports that both *sxtA* gene motifs, *sxtA1* and *sxtA4*, exist in the STX-producing dinoflagellates analyzed, but they are absent in non-toxic species except for several supposed non-STX-producing strains [21]. However, sequences of both motifs were found to be present in the genomes of ACHK-T and ACHK-NT (see Supplementary Information). Even if the *sxtA4* motif still remained, it was not transcribed in ACHK-NT from the RNA-seq results as well as qPCR analysis. This implied that mutations might occur in the *sxtA* of the non-toxic strain, resulting in the silence of the gene for the initiation of STX biosynthesis. In addition, the expression of the shorter transcript of *sxtA* was the same as that in ACHK-T, indicating that mutation was more likely to be located at the genomic sequence of the *sxtA4* motif. We attempted to amplify the whole coding sequence of *sxtA4* to explore the potential mutation sites but failed owing to the complexity of the sequence containing too many SNPs [21]. The performance of *sxtA* in toxic *A. catenella* and its non-toxic mutant implied that *sxtA*, especially *sxtA4,* might be essential for toxin biosynthesis, in accordance with previous reported result that the C-terminal of sxtA is critical for STX biosynthesis [23]. In addition, three unigenes (comp66059_c0, comp71917_c0, comp64901_c0) which were assigned to splicing factor related genes were found to be differentially expressed between ACHK-NT and ACHK-T (Supplementary Table S1). Mutations of these genes might also result in the down-regulation of *sxtA* in ACHK-NT, and subsequently the loss of the ability to produce toxins. 

### 3.3. STX Biosynthesis Related Genes

The two searching strategies we applied identified a total of 101 homologs of 12 cyanobacterial *sxt* genes, including most of the core toxin genes (Figure 3). However, no significant alignment results were obtained based on the BLASTN approach, even for the characterized *sxtA* and *sxtG* genes of dinoflagellates. The enormous discrepancies of nucleotide sequences between *Alexandrium* spp. and cyanobacteria might be explained by vast variations of genes after transfer into dinoflagellate genomes or the possible independent evolution of the STX biosynthetic pathway in these two organisms [16,21,22]. Nevertheless, identification based on similarity of amino acid sequences produced all the homologs, indicating that their encoding genes potentially participated in STX biosynthesis. Except for the ten toxin genes reported, *sxtO* and *sxtZ* were identified in dinoflagellates for the first time (Table 4, Figure 3). *sxtO* is involved in the conversion of STX analogs, encoding an adenylylsulfate kinase which catalyzed the conversion of adenylylsulfate to 3′-phosphate 5′-phosphosulfate (PAPS), the sulfate donor for PAPS dependent sulfotransferases encoded by *sxtN*. Expression of *sxtO* was also supported by the existence of C1/C2 and GTX1/4 toxin in the detectable toxin composition of ACHK-T. *sxtZ* is believed to regulate STX production at transcriptional level in cyanobacteria and, therefore, detection of *sxtZ* suggested that toxin production might be regulated in the same manner in dinoflagellates. The remainder of the toxin biosynthesis genes was not found in our study using either searching method. This might have resulted from homolog losses of these genes and functional substitutions by other genes in dinoflagellates [16]. Among the DEGs between the two samples, several unigenes were annotated as hypothetical proteins or without NR annotation. We considered these genes as *sxt* gene candidates that might be directly or indirectly involved in STX biosynthesis, especially for those down-regulated genes, and their depression possibly resulted in the loss of ability to produce toxin in ACHK-NT. To confirm the exact functions of these genes, more functional investigations still need to be carried out.

## 4. Experimental Section

### 4.1. Organisms and Culture Conditions

The toxic *Alexandrium catenella* (ACHK-T) and its non-toxic mutant strain (ACHK-NT) were provided by the Collection Center of Marine Bacteria and Algae, Xiamen University, Xiamen, China. Cultures were maintained in K medium [47] at 20 °C under a 14:10 h light: dark photoperiod at a light intensity of approximately 100 μmol photons m^−2^·s^−1^ provided by fluorescent lamps. Before RNA-seq analysis, 18S sequence (accession numbers: KM091275, KM091276) and toxin composition of two strains were examined, and the results were consistent with the previously published results (Supplementary Figure S1) [26].

### 4.2. Sample Collection for Cell Density, Toxin Measurement and Cell Cycle Analysis

To determine an optimal time point for transcriptome comparison, sampling was carried out every 2 h from the onset of the light period (T1, 08:00) to the end (T8, 22:00). At each time point, 100 mL of ACHK-T culture was collected and 0.5 mL of 50 mM acetic acid was added to suspend the cell pellets. Toxin was extracted and subsequently measured with HPLC using an Inertsil C8-5 column (15 cm × 4.6 cm, GL Science, Tokyo, Japan) as reported previously [26]. Two dominant PSP toxins, C1/C2 and GTX were examined. At the same sampling time, 50 mL of each culture was collected for cell cycle analysis using the previously reported method [25]. In addition, three 1 mL of each culture were collected every 2 h and fixed with Lugol’s solution for subsequent cell counting.

### 4.3. RNA Isolation, cDNA Library Preparation and Sequencing

Each of the two strains was cultured in triplicate with the same types of flasks. During the exponential growth phase, cells were harvested at T5 (16:00) based on the toxin measurement results. Collection of samples was conducted as described above. The pellets were then resuspended with 1 mL Trizol Reagent (Invitrogen, Carlsbad, CA, USA), immediately frozen in liquid nitrogen and subsequently stored at −80 °C for later RNA extraction.

Total RNA was isolated using TRI-Reagent (MRC, Cincinnati, OH, USA). Degradation and contamination of RNA was monitored on 1% agarose gels. RNA concentration was measured using the Qubit^®^ RNA Assay Kit in a Qubit^®^ 2.0 Flurometer (Life Technologies, Carlsbad, CA, USA). RNA integrity was assessed using the RNA Nano 6000 Assay Kit of the Bioanalyzer 2100 system (Agilent Technologies, Santa Clara, CA, USA).

Sequencing libraries were generated using the Illumina TruSeq™ RNA Sample Preparation Kit (Illumina, San Diego, CA, USA) following the manufacturer’s instructions. Poly-T oligo-attached magnetic beads were used to enrich poly-A mRNA, followed by fragmentation. First strand cDNA was synthesized using random oligonucleotides and SuperScript II (Life Technologies, Carlsbad, CA, USA). Second strand cDNA synthesis was subsequently performed using DNA Polymerase I and RNase H. After conversion of the remaining overhangs into blunt ends and ligation of adapters, cDNA fragments of preferentially 200 bp in length were selected to be enriched in a 10 cycle PCR reaction. Products were purified (AMPure XP system, Beckman Coulter, Beverly, MA, USA) and quantified using the Agilent high sensitivity DNA assay on the Agilent Bioanalyzer 2100 system (Santa Clara, CA, USA). Subsequently, the library preparations were sequenced using Illumina Hiseq^TM^ 2000 (San Diego, CA, USA).

### 4.4. Quality Control and de novo Assembly

Raw data (raw reads) of fastq format were first processed through custom Perl scripts. Clean data (clean reads) were obtained by removing reads containing adapter, reads containing poly-N, and low quality reads from the raw data, and the Q20 of the clean data were calculated. All the downstream analyses were based on clean data with high quality. The left read files from both libraries were pooled into one left.fq file and the right read files into one right.fq file. *De novo* assembly was accomplished based on the left.fq and right.fq using Trinity with min_kmer_cov set to 2 and all other parameters set to default [28]. To avoid the redundant transcripts, in-house Perl scripts were applied to extract the longest transcripts as unigenes. Unigenes generated with the assembly were used for downstream analysis.

### 4.5. Gene Functional Annotation and Differential Expression Analysis

The unigenes were searched against the NCBI NR database, NCBI NT database, KOG and Swiss-Prot database using BLAST (release 2.2.27) with a threshold e-value ≤ 1e^−5^. The best hit with the lowest e-value was assigned for each unigene annotation. Based on the NR annotation, Blast2GO (version 2.5, http://www.blast2go.com/b2ghome) software was applied to perform GO terms annotation and functional classification. Using annotated GO terms, enzyme codes were assigned to unigenes with EC numbers and KEGG pathways. Using the KAAS annotation (http://www.genome.jp/tools/kaas/), KO codes were obtained for each unigene. Moreover, to provide potential functional information based on the conserved domain of genes, coding region sequences were predicted. The ORF of unigenes was determined with results of BLAST against protein databases in the priority order of NR, Swiss-Prot and KEGG. With the standard codon table, the coding region sequences of unigenes were then translated into amino acid sequences. For unigenes with no hit against protein databases, potential ORF was predicted using the ESTScan program and translation was conducted. Utilizing HMMER 3.0 (http://hmmer.janelia.org/), amino acid sequences were submitted to search the Pfam database for functional domain and Pfam annotation of unigenes.

To calculate gene expression level, transcripts assembled with Trinity were used as reference sequences. Using RSEM (), clean reads of each sample were mapped back to the reference sequences and read counts of transcripts were obtained, followed by normalization of read count number to RPKM [30]. Differential expression analysis of genes from the two samples was performed using the DEGseq (2010) R package [48]. The *p*-values were adjusted to be *q*-values as reported previously [49]. A fold change ≥ 1.5 and a *q*-value < 0.05 were set as the threshold for significant differential expression.

### 4.6. Identification of STX Genes

Except for *sxtA* and *sxtG* which have been identified, the sequences and exact functions of the other genes related to STX synthesis in dinoflagellates are still inconclusive. To ascertain genes potentially related to STX synthesis in dinoflagellates, BLAST was performed with known cyanobacteria *sxt* genes against unigenes datasets in this study. A search strategy based on either nucleotide or amino acid sequences was attempted as reported [21,23]. Cyanobacterial *sxt* genes and all 26 proteins encoded by the *sxt* cluster were used as queries to seek homologs with an e-value < 0.1 (BLASTN) and < 1e^−5^ (TBLASTN).

### 4.7. qRT-PCR Validation of DEGs

Total RNA was extracted as described above from the same samples that were used in the RNA-seq analysis. The RNA of each sample was treated with RNase-free DNase I to (Promega, Madison, WI, USA) remove genomic DNA and then 200 ng treated RNA was subjected to first-strand cDNA synthesis with oligo-dT primers and MMLV transcriptase (Promega, Madison, WI, USA). qRT-PCR was performed on a Bio-Rad CFX96 real-time PCR detection system (Bio-Rad, Hercules, CA, USA) using iQ SYBR Green Supermix (Bio-Rad, Hercules, CA, USA). The thermo cycle was: 95 °C for 30 s, 40 cycles at 94 °C for 15 s, and 58 °C for 30 s. Dinoflagellate *calmodulin* was chosen as the reference gene to normalize expression level. Primers used for qPCR analysis were listed in Supplementary Table S3 [50]. Relative change of gene expression was calculated with the 2^−ΔΔCt^ method as reported.

## 5. Conclusions

This study compared transcriptomic profiles of a toxigenic *A. catenella* and its non-toxigenic mutant using RNA-seq. Gene expression patterns of the two strains were highly similar, but 35 genes presented differential expressions. These genes were mainly involved in photosynthesis, carbon fixation and amino acid metabolism as well as toxin biosynthesis. 101 homologs of 12 cyanobacterial *sxt* genes, including most of the core toxin genes were identified. Among them, the long transcript of the *sxtA* gene directly participating in STX biosynthesis was depressed significantly which might terminate the first step of the toxin biosynthesis pathway, resulting in the loss of ability to produce toxin in the mutant ACHK-NT. Compared with the long transcript, the short one lack of *sxtA4* domain presented similar expressions between the two strains, indicating *sxtA4* might be critical for toxin biosynthesis. In addition to genes directly implicated in STX biosynthesis, putative *sxtO* and *sxtZ* functioning as conversion of STX analogs and transcriptional regulation of STX production were identified for the first time in dinoflagellates. Transcriptome data from this study suggested that more carbon and energy were utilized for cell growth in ACHK-NT, and mutation of the *sxtA4* motif might be responsible for the loss of ability to produce toxin. However, the exact mutation localization in the genomic sequence of this motif needs further study.

## Supplementary Files

Supplementary File 1

Supplementary File 2

## References

[1] Wiese M., DʼAgostino P.M., Mihali T.K., Moffitt M.C., Neilan B.A. (2010). Neurotoxic alkaloids: Saxitoxin and its analogs. Mar. Drugs.

[2] Hallegraeff G.M., Hallegraeff G.M., Anderson D.M., Cembella A.D. (1995). Harmful algal blooms: A global overview. Manual on Harmful Marine Microalgae.

[3] Cembella A.D., Hallegraeff G.M., Anderson D.M., Cembella A.D. (1998). Ecophysiology and metabolism of paralytic shellfish toxins in marine microalgae. Physiological Ecology of Harmful Algal Blooms.

[4] Orr R.J.S., Stüken A., Rundberget T., Eikrem W., Jakobsen K.S. (2011). Improved phylogenetic resolution of toxic and non-toxic *Alexandrium* strains using a concatenated rDNA approach. Harmful Algae.

[5] Sivonen K., Jones G., Chorus I., Bartram J. (1999). Cyanobacterial Toxins. Toxic Cyanobacteria in Water: A Guide to Their Public Health Consequences, Monitoring and Management.

[6] Chorus I., Falconer I.R., Salas H.J., Bartram J. (2000). Health risks caused by freshwater cyanobacteria in recreational waters. J. Oxicol. Environ. Health. Part. B Crit. Rev..

[7] Carmichael W.W. (2001). Health Effects of Toxin-Producing Cyanobacteria: “The CyanoHABs”. Hum. Ecol. Risk Assess..

[8] Haider S., Naithani V., Viswanathan P.N., Kakkar P. (2003). Cyanobacterial toxins: A growing environmental concern. Chemosphere.

[9] Kellmann R., Mihali T.K., Jeon Y.J., Pickford R., Pomati F., Neilan B.A. (2008). Biosynthetic intermediate analysis and functional homology reveal a saxitoxin gene cluster in cyanobacteria. Appl. Environ. Microbiol..

[10] Mihali T.K., Kellmann R., Neilan B.A. (2009). Characterisation of the paralytic shellfish toxin biosynthesis gene clusters in *Anabaena circinalis* AWQC131C and *Aphanizomenon* sp. NH-5. BMC Biochem..

[11] Mihali T.K., Carmichael W.W., Neilan B.A. (2011). A putative gene cluster from a *Lyngbya wollei* bloom that encodes paralytic shellfish toxin biosynthesis. PLoS One.

[12] Stucken K., John U., Cembella A., Murillo A.A., Soto-Liebe K., Fuentes-Valdés J.J., Friedel M., Plominsky A.M., Vásquez M., Glöckner G. (2010). The Smallest Known Genomes of Multicellular and Toxic Cyanobacteria: Comparison, Minimal Gene Sets for Linked Traits and the Evolutionary Implications. PLoS One.

[13] Shimizu Y. (1996). Microalgal metabolites: A new perspective. Annu. Rev. Microbiol..

[14] Kellmann R., Stuken A., Orr R.J., Svendsen H.M., Jakobsen K.S. (2010). Biosynthesis and molecular genetics of polyketides in marine dinoflagellates. Mar. Drugs.

[15] Kellmann R., Neilan B.A. (2007). Biochemical Characterization of Paralytic Shellfish Toxin Biosynthesis *in Vitro*. J. Phycol..

[16] Orr R.J., Stuken A., Murray S.A., Jakobsen K.S. (2013). Evolution and distribution of saxitoxin biosynthesis in dinoflagellates. Mar. Drugs.

[17] Li L., Hong R., Hastings J.W. (1997). Three functional luciferase domains in a single polypeptide chain. Proc. Natl. Acad. Sci. USA.

[18] LaJeunesse T.C., Lambert G., Andersen R.A., Coffroth M.A., Galbraith D.W. (2005). *Symbiodinium* (pyrrhophyta) genome sizes (DNA content) are smallest among dinoflagellates. J. Phycol..

[19] Harlow L.D., Koutoulis A., Hallegraeff G.M. (2007). S-adenosylmethionine synthetase genes from eleven marine dinoflagellates. Phycologia.

[20] Yang I., John U., Beszteri S., Glockner G., Krock B., Goesmann A., Cembella A.D. (2010). Comparative gene expression in toxic *versus* non-toxic strains of the marine dinoflagellate *Alexandrium minutum*. BMC Genomics.

[21] Stuken A., Orr R.J., Kellmann R., Murray S.A., Neilan B.A., Jakobsen K.S. (2011). Discovery of nuclear-encoded genes for the neurotoxin saxitoxin in dinoflagellates. PLoS One.

[22] Orr R.J., Stuken A., Murray S.A., Jakobsen K.S. (2013). Evolutionary Acquisition and Loss of Saxitoxin Biosynthesis in Dinoflagellates: the Second “Core” Gene, sxtG. Appl. Environ. Microbiol..

[23] Hackett J.D., Wisecaver J.H., Brosnahan M.L., Kulis D.M., Anderson D.M., Bhattacharya D., Plumley F.G., Erdner D.L. (2013). Evolution of saxitoxin synthesis in cyanobacteria and dinoflagellates. Mol. Biol. Evol..

[24] Cho Y., Hiramatsu K., Ogawa M., Omura T., Ishimaru T., Oshima Y. (2008). Non-toxic and toxic subclones obtained from a toxic clonal culture of *Alexandrium tamarense* (Dinophyceae): Toxicity and molecular biological feature. Harmful Algae.

[25] Wang D.Z., Gao Y., Lin L., Hong H.S. (2013). Comparative proteomic analysis reveals proteins putatively involved in toxin biosynthesis in the marine dinoflagellate *Alexandrium catenella*. Mar. Drugs.

[26] Wang D.Z., Li C., Zhang Y., Wang Y.Y., He Z.P., Lin L., Hong H.S. (2012). Quantitative proteomic analysis of differentially expressed proteins in the toxicity-lost mutant of *Alexandrium catenella* (Dinophyceae) in the exponential phase. J. Proteomics.

[27] Cho Y., Ogawa M., Yotsu-Yamashita M., Oshima Y. (2014). Effect of 5-fluoro-2′-deoxyuridine on toxin production and cell cycle regulation in marine dinoflagellate, *Alexandrium tamarense*. Harmful Algae.

[28] Grabherr M.G., Haas B.J., Yassour M., Levin J.Z., Thompson D.A., Amit I., Adiconis X., Fan L., Raychowdhury R., Zeng Q. (2011). Full-length transcriptome assembly from RNA-Seq data without a reference genome. Nat. Biotechnol..

[29] Zhang S., Sui Z., Chang L., Kang K., Ma J., Kong F., Zhou W., Wang J., Guo L., Geng H. (2014). Transcriptome de novo assembly sequencing and analysis of the toxic dinoflagellate *Alexandrium catenella* using the Illumina Platform. Gene.

[30] Li B., Dewey C.N. (2011). RSEM: Accurate transcript quantification from RNA-Seq data with or without a reference genome. BMC Bioinfor..

[31] Robinson M.D., Oshlack A. (2010). A scaling normalization method for differential expression analysis of RNA-seq data. Genome Biol..

[32] Haiser H.J., Yousef M.R., Elliot M.A. (2009). Cell wall hydrolases affect germination, vegetative growth, and sporulation in *Streptomyces coelicolor*. J. Bacteriol..

[33] Murray S.A., Mihali T.K., Neilan B.A. (2011). Extraordinary conservation, gene loss, and positive selection in the evolution of an ancient neurotoxin. Mol. Biol. Evol..

[34] Haas B.J., Papanicolaou A., Yassour M., Grabherr M., Blood P.D., Bowden J., Couger M.B., Eccles D., Li B., Lieber M. (2013). *De novo* transcript sequence reconstruction from RNA-seq using the Trinity platform for reference generation and analysis. Nat. Protoc..

[35] Hou Y., Lin S. (2009). Distinct gene number-genome size relationships for eukaryotes and non-eukaryotes: Gene content estimation for dinoflagellate genomes. PLoS One.

[36] Murray S.A., Wiese M., Stuken A., Brett S., Kellmann R., Hallegraeff G., Neilan B.A. (2011). *sxtA*-based quantitative molecular assay to identify saxitoxin-producing harmful algal blooms in marine waters. Appl. Environ. Microbiol..

[37] Romeis T., Ludwig A.A., Martin R., Jones J.D. (2001). Calcium-dependent protein kinases play an essential role in a plant defence response. EMBO J..

[38] Cramer G.R., Van Sluyter S.C., Hopper D.W., Pascovici D., Keighley T., Haynes P.A. (2013). Proteomic analysis indicates massive changes in metabolism prior to the inhibition of growth and photosynthesis of grapevine (*Vitis vinifera* L.) in response to water deficit. BMC Plant. Biol..

[39] Taroncher-Oldenburg G., Anderson D.M. (2000). Identification and characterization of three differentially expressed genes, encoding *S*-adenosylhomocysteine hydrolase, methionine aminopeptidase, and a histone-like protein, in the toxic dinoflagellate *Alexandrium fundyense*. Appl. Environ. Microbiol..

[40] Takahashi M., Takahashi H., Nakano Y., Konishi T., Terauchi R., Takeda T. (2010). Characterization of a cellobiohydrolase (MoCel6A) produced by *Magnaporthe oryzae*. Appl. Environ. Microbiol..

[41] Hackett J.D., Anderson D.M., Erdner D.L., Bhattacharya D. (2004). Dinoflagellates: A remarkable evolutionary experiment. Am. J. Bot..

[42] Kwok A.C., Wong J.T. (2003). Cellulose synthesis is coupled to cell cycle progression at G1 in the dinoflagellate *Crypthecodinium cohnii*. Plant. Physiol..

[43] Kwok A.C., Wong J.T. (2010). The activity of a wall-bound cellulase is required for and is coupled to cell cycle progression in the dinoflagellate *Crypthecodinium cohnii*. Plant. Cell Online.

[44] Toulza E., Shin M.S., Blanc G., Audic S., Laabir M., Collos Y., Claverie J.M., Grzebyk D. (2010). Gene expression in proliferating cells of the dinoflagellate *Alexandrium catenella* (Dinophyceae). Appl. Environ. Microbiol..

[45] Radmer R., Cox J., Lieberman D., Behrens P., Arnett K. (1987). Biomass recycle as a means to improve the energy efficiency of CELSS algal culture systems. Adv. Space Res..

[46] Blifernez-Klassen O., Klassen V., Doebbe A., Kersting K., Grimm P., Wobbe L., Kruse O. (2012). Cellulose degradation and assimilation by the unicellular phototrophic eukaryote *Chlamydomonas reinhardtii*. Nat. Commun..

[47] Keller M.D., Selvin R.C., Claus W., Guillard R.R. (1987). Media for the culture of oceanic ultraphytoplankton. J. Phycol..

[48] Wang L., Feng Z., Wang X., Wang X., Zhang X. (2009). DEGseq: An R package for identifying differentially expressed genes from RNA-seq data. Bioinformatics.

[49] Storey J.D., Tibshirani R. (2003). Statistical significance for genomewide studies. Proc. Natl. Acad. Sci. USA.

[50] Zhang H., Lin S. (2003). Complex gene structure of the form ii rubisco in the dinoflagellate *Prorocentrum minimum* (dinophyceae). J. Phycol..

