# Supplementary material for: Comparative Transcriptome Analysis of a Toxin-Producing Dinoflagellate Alexandrium catenella and Its Non-Toxic Mutant
Source: Mar Drugs. 2014 Nov 24;12(11):5698–718. doi: 10.3390/md12115698 (PMC4245552; doi:10.3390/md12115698)
Supplement: Supplementary File 2 [file marinedrugs-12-05698-s002.pdf]

Supplementary Information

Figure S1. High performance liquid chromatography of STX in ACHK-T and ACHK-NT. (**A**) C1/C2 toxins; (**B**) GTX1/4 toxins. Y-axis: Fluorescence intensity (LU); X-axis: Retention time (min). Differences of chromatograms peak between ACHK-T and ACHK-NT were consistent with previous result [26].

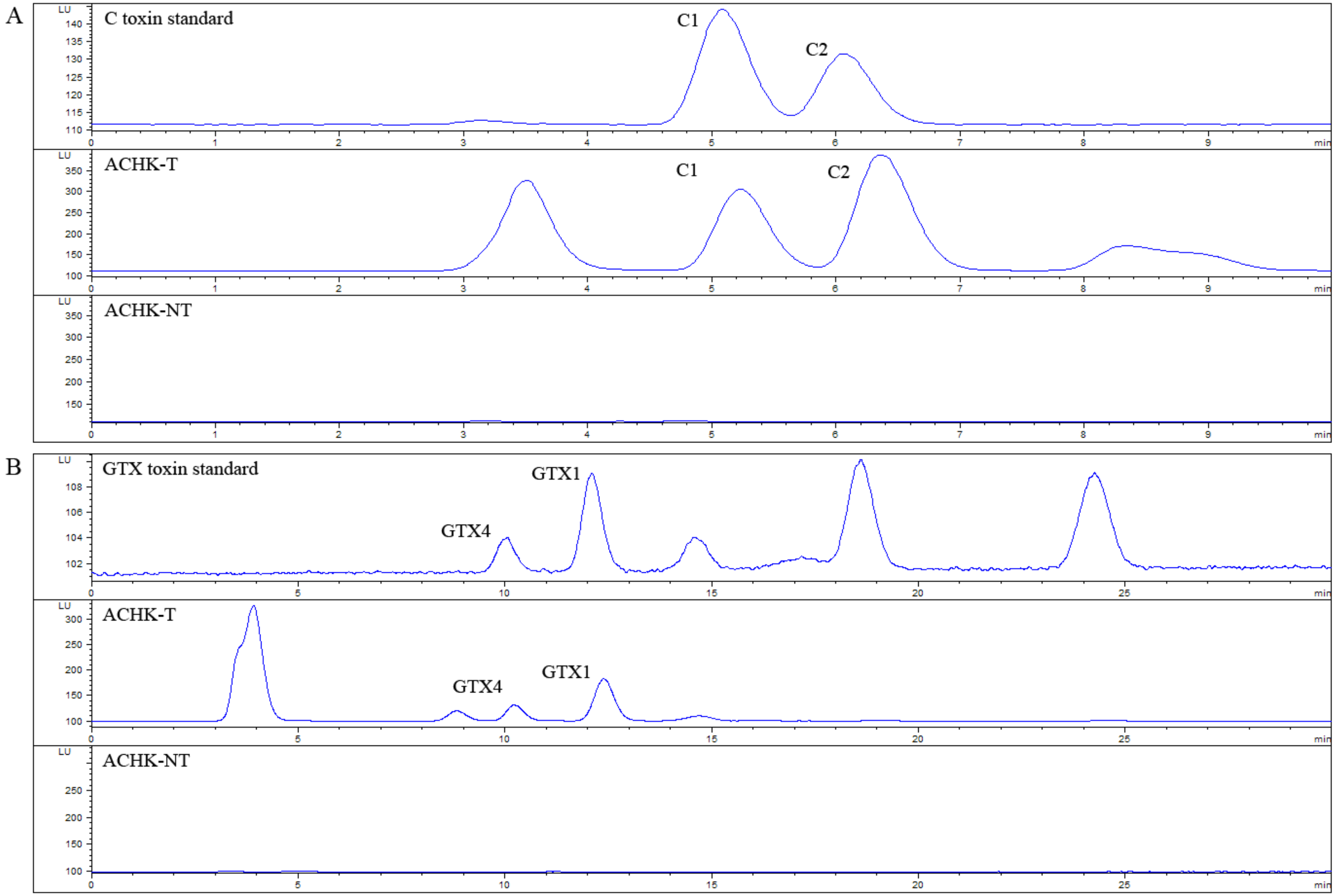

Figure S2. Cell cycle phase distribution of ACHK-T and ACHK-NT during the light period. Y-axis: Number of cells; X-axis: the relative amount of DNA. The results presented a similar phase distributions between the two strains.

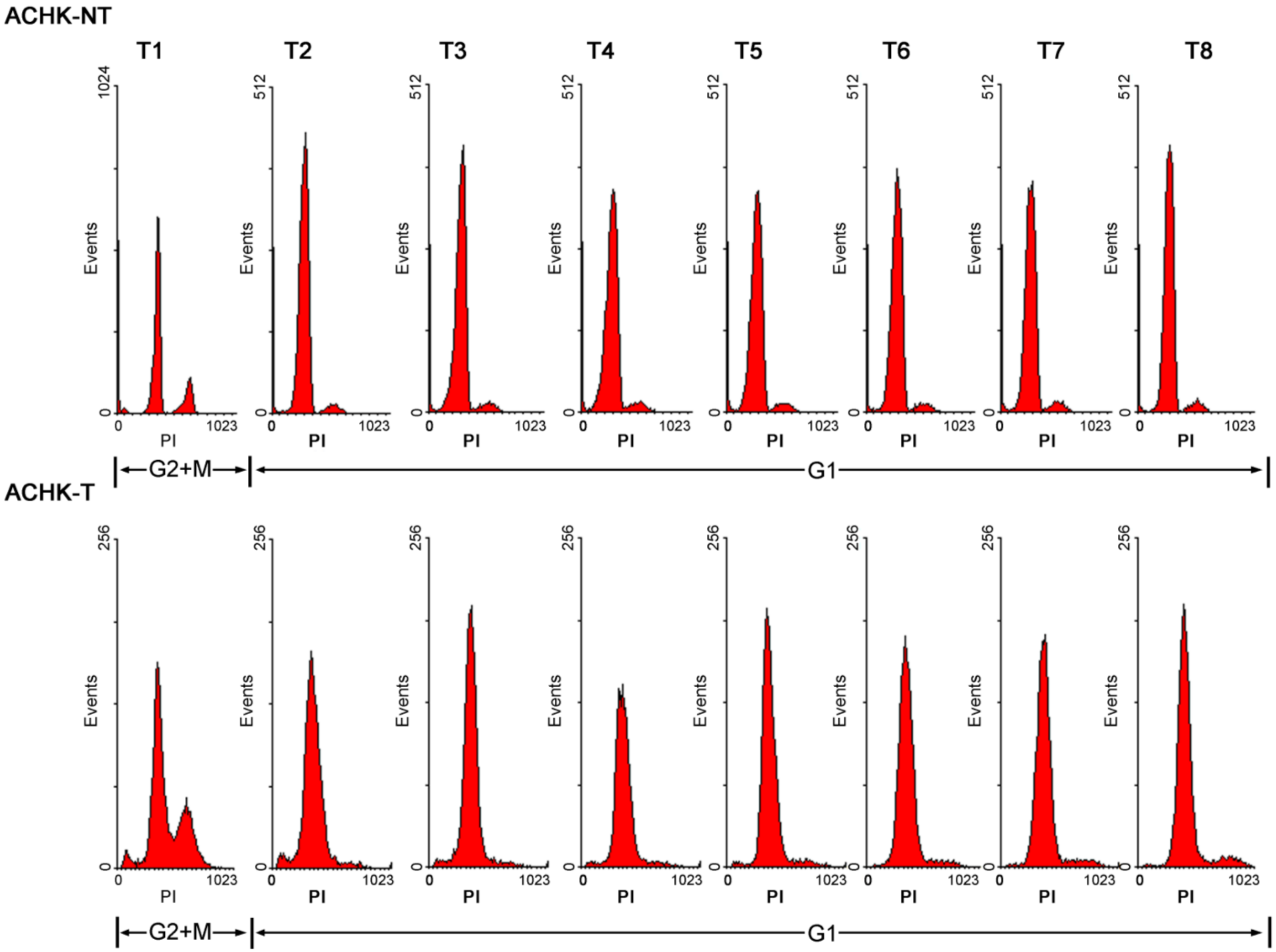

Figure S3. Uniform distribution of all reads from libraries of ACHK-NT and ACHK-T. Homogeneous distribution of reads which covered multiple locations in UTRs and coding regions of transcripts showed an evenly distributed coverage of reads to genes.

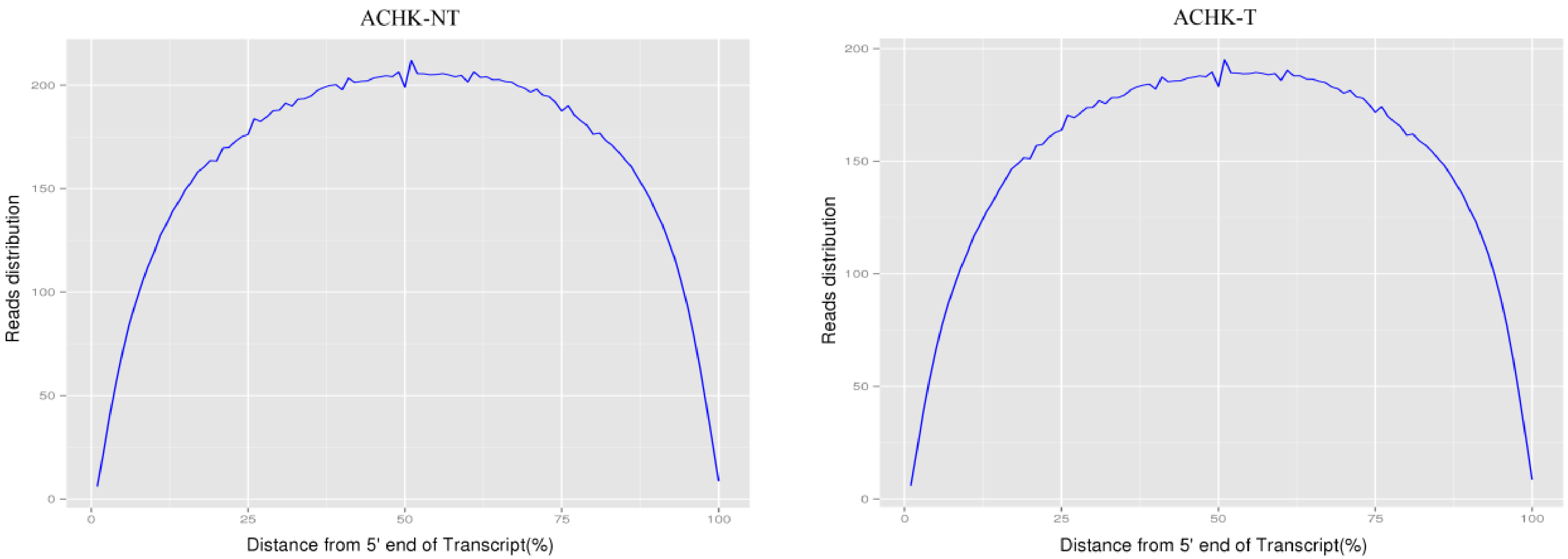

Figure S4. KOG classification of *A. catenella* unigenes. Letters represent the 26 classifications in KOG databases.

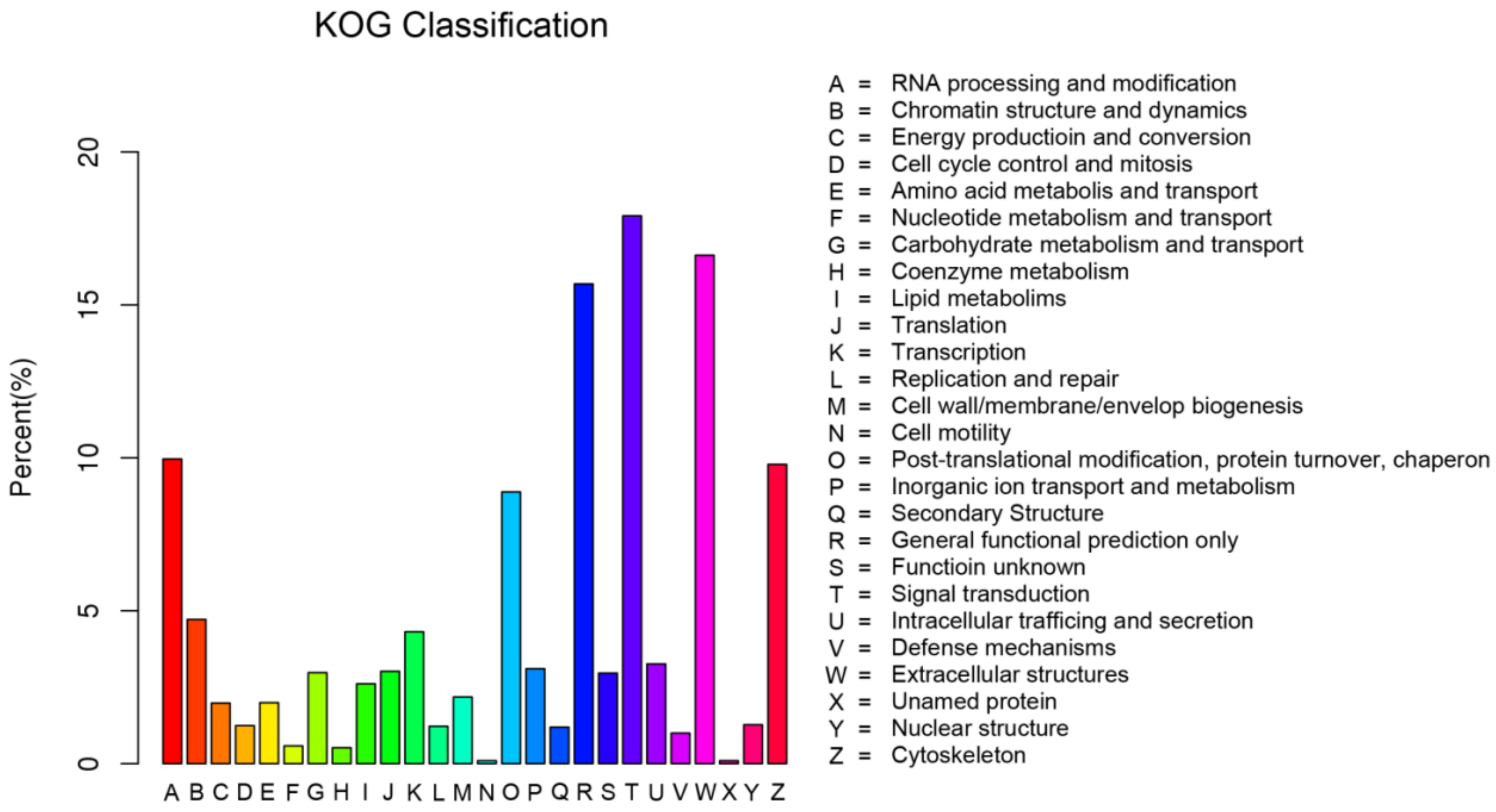

Figure S5. GO classification of *A. catenella* unigenes. Each annotated sequence was assigned to at least one GO term and classified into different groups within the three functional categories.

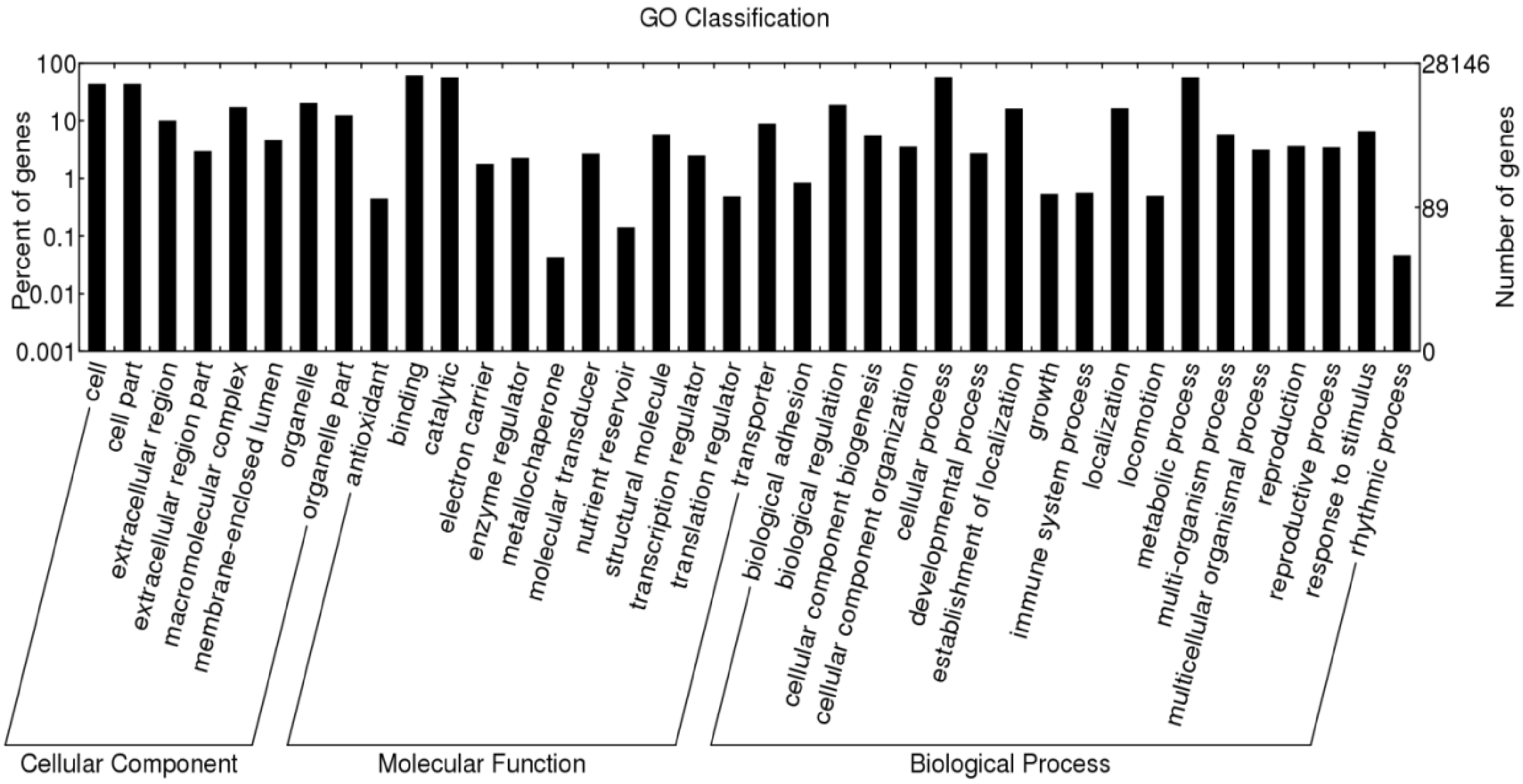

Figure S6. KEGG pathway annotation and KO annotation. (**A**) Top 10 represented KEGG pathway. Number of unigenes and EC numbers involved in each pathway is shown; (**B**) Top 10 KO terms with number of unigenes assigned to each term.

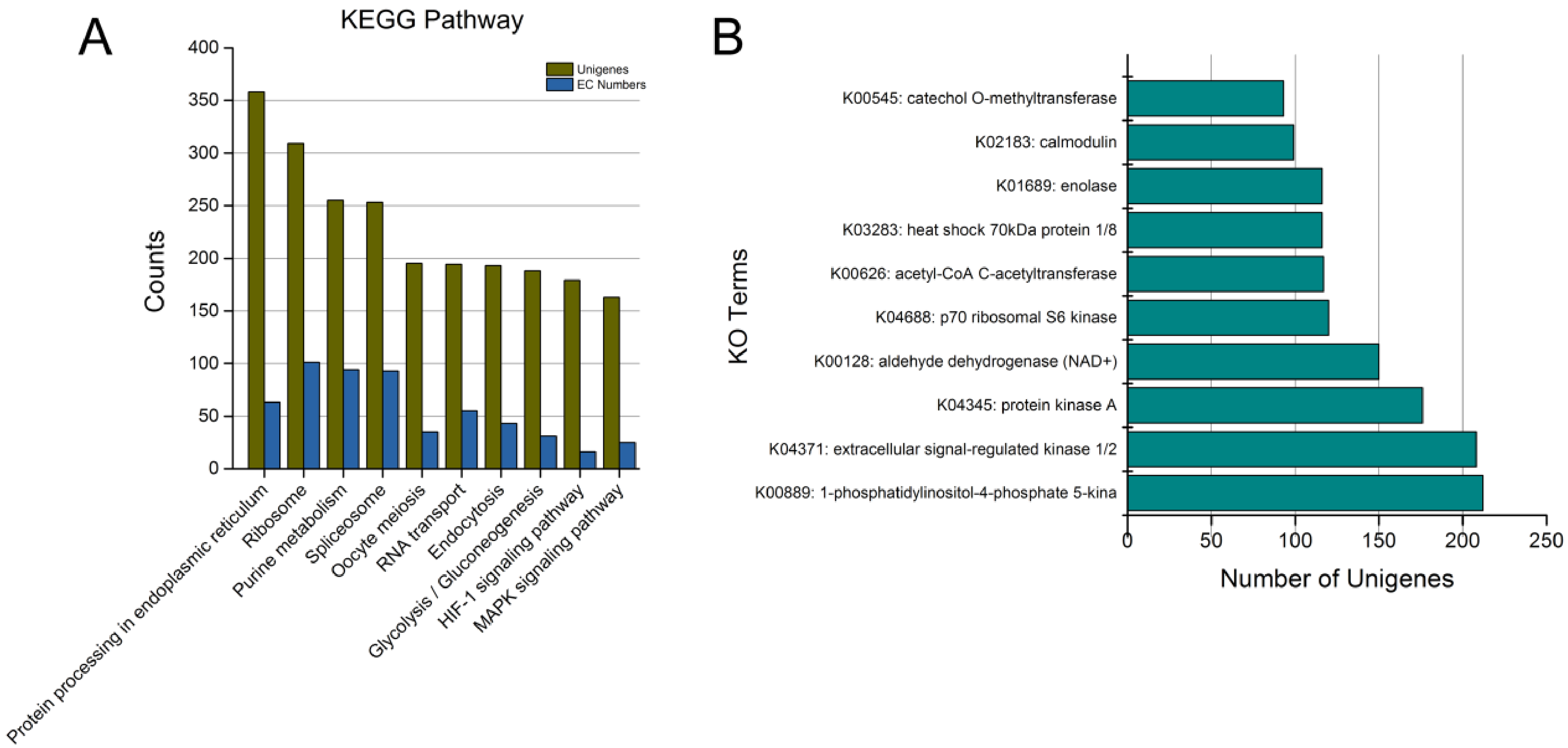

Figure S7. KEGG classifications of unigenes based on the secondary pathway hierarchy. Letters A to D represent the four primary hierarchies in KEGG database: **A**: Cellular Processes; **B**: Environmental Information Processing; **C**: Genetic Information Processing; **D**: Metabolism.

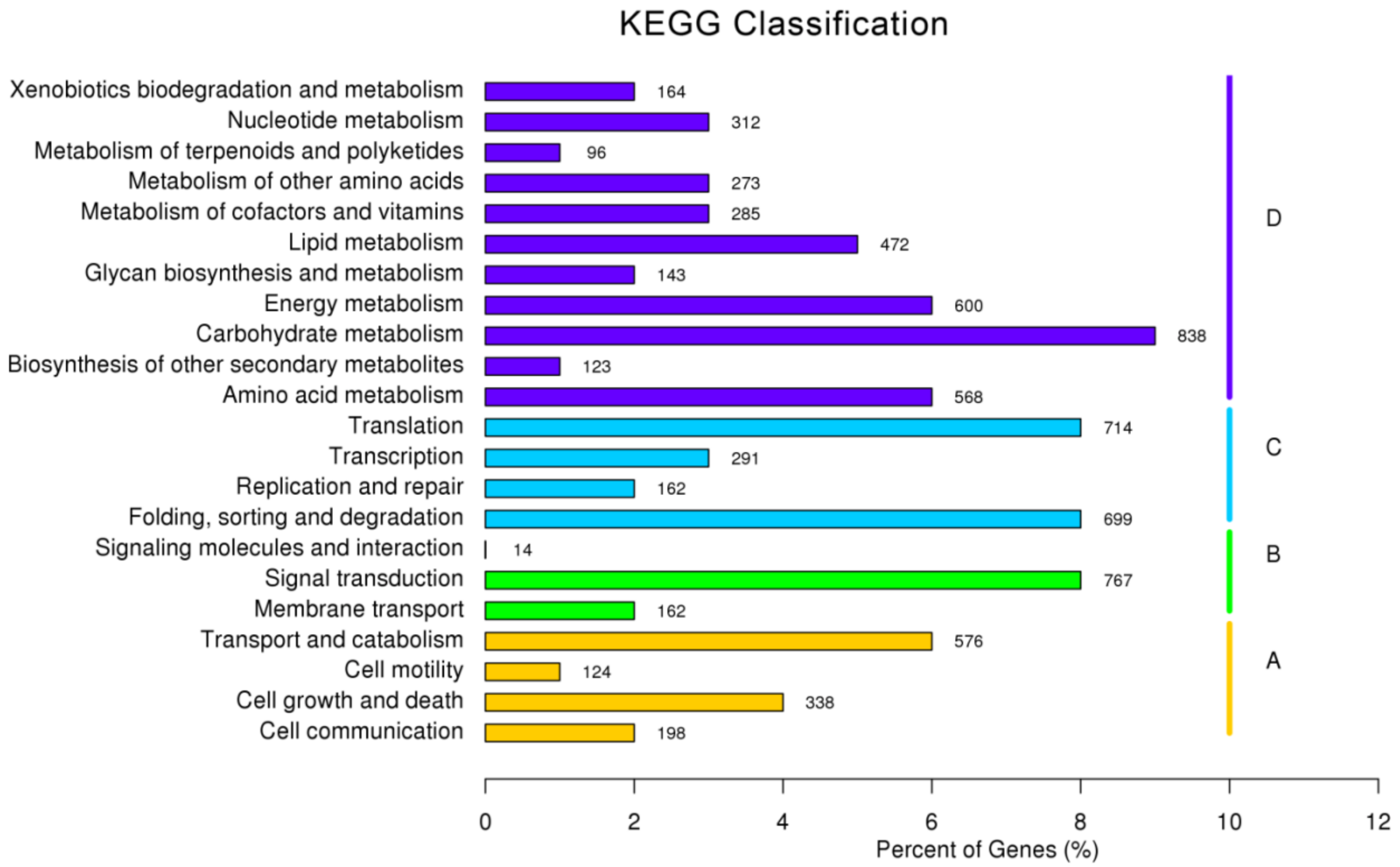

Figure S8. Protein families in *A. catenella* transcriptome. (**A**) Distribution of Pfam families versus unigenes; (**B**) Top 10 represented Pfam families.

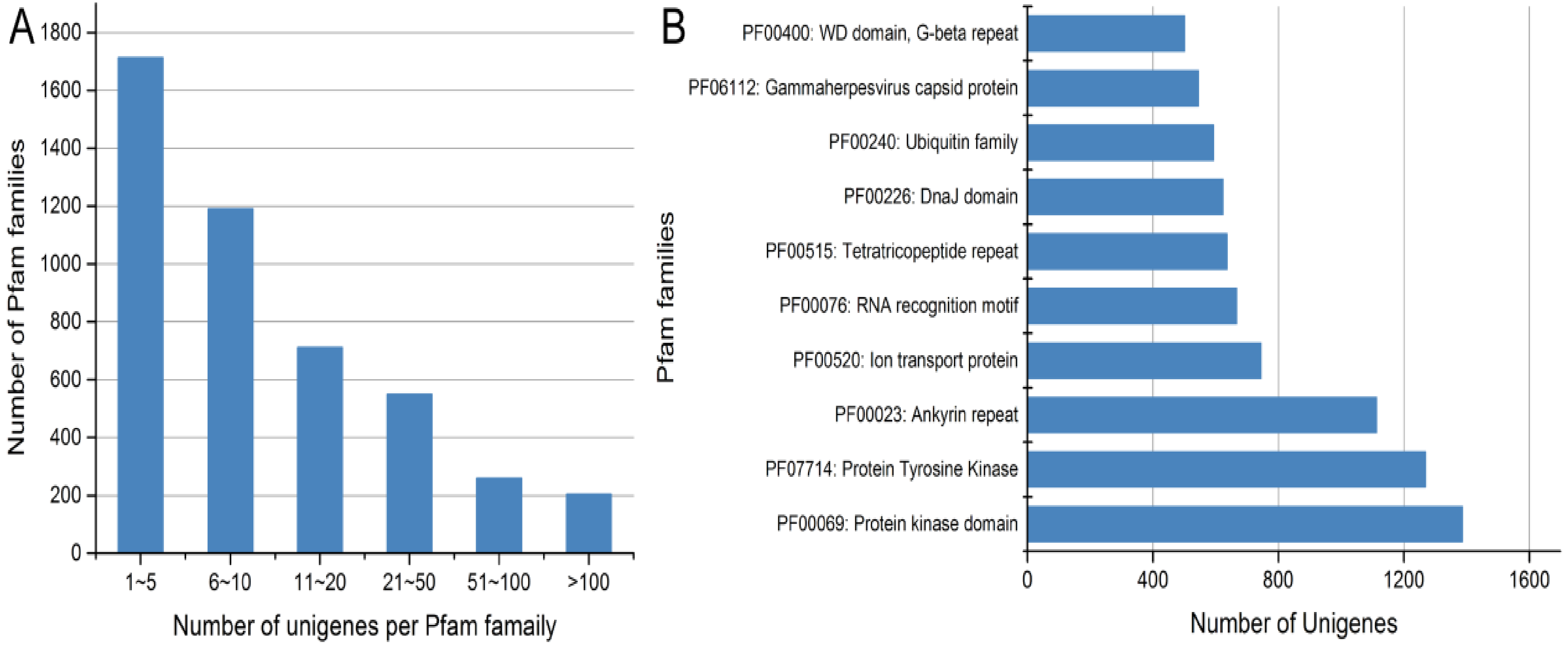

Figure S9. Alignment and GC content comparison of both isoforms of *sxtA* obtained in this study with reported complete cds sequences of *sxtA* from *A. fundyense*. (**A**) Alignment of comp66169 with *sxtA* long transcript (accession number: JF343239); (**B**) Alignment of comp20666 with *sxtA* short transcript (accession number: JF343238). The color labeled nucleotides were DinoSL and polyA regions which were not obtained in this study; (**C**) GC content comparison. GC content was calculated using the reported method [21].

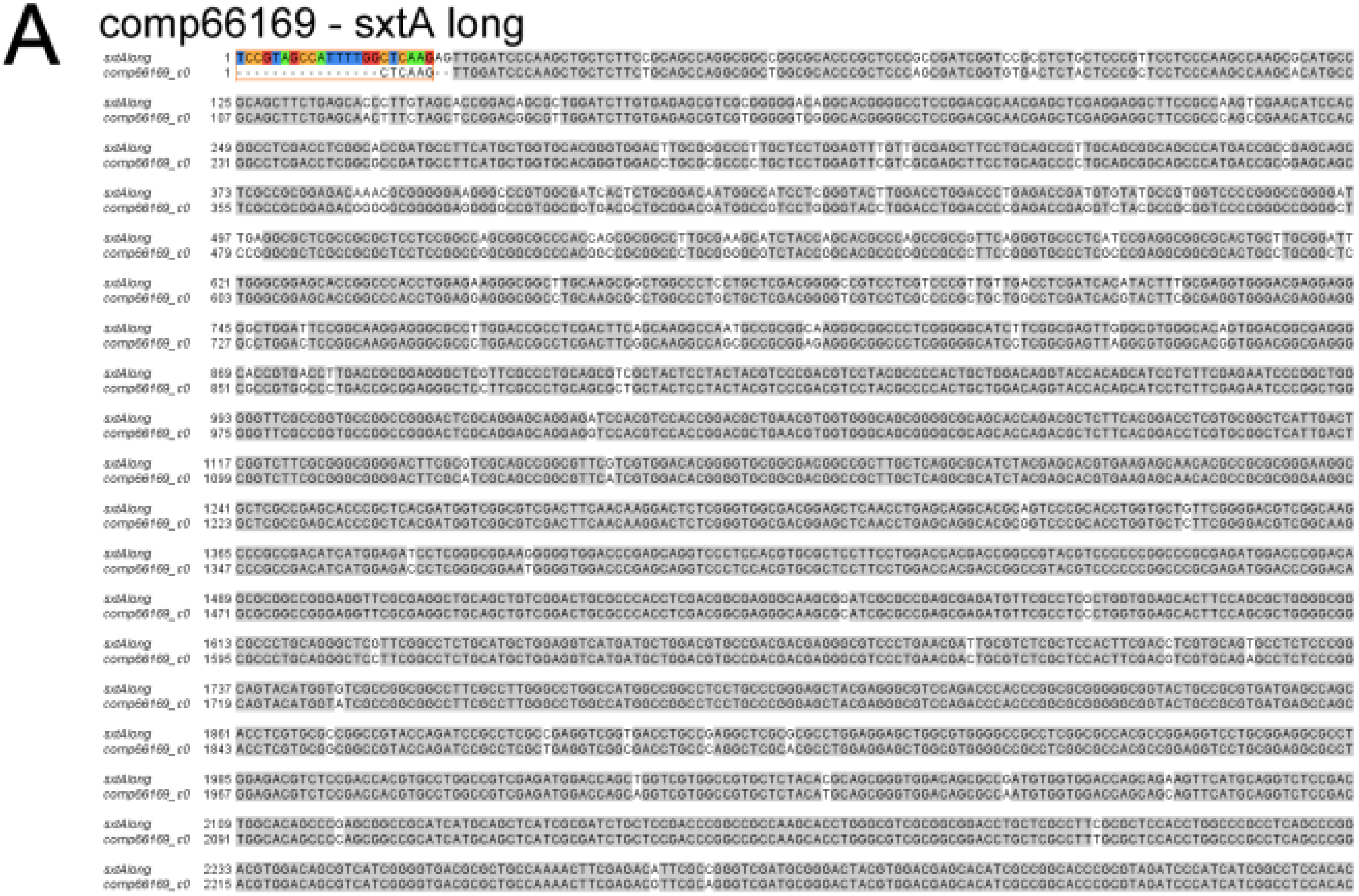

Figure S9. *Cont.*

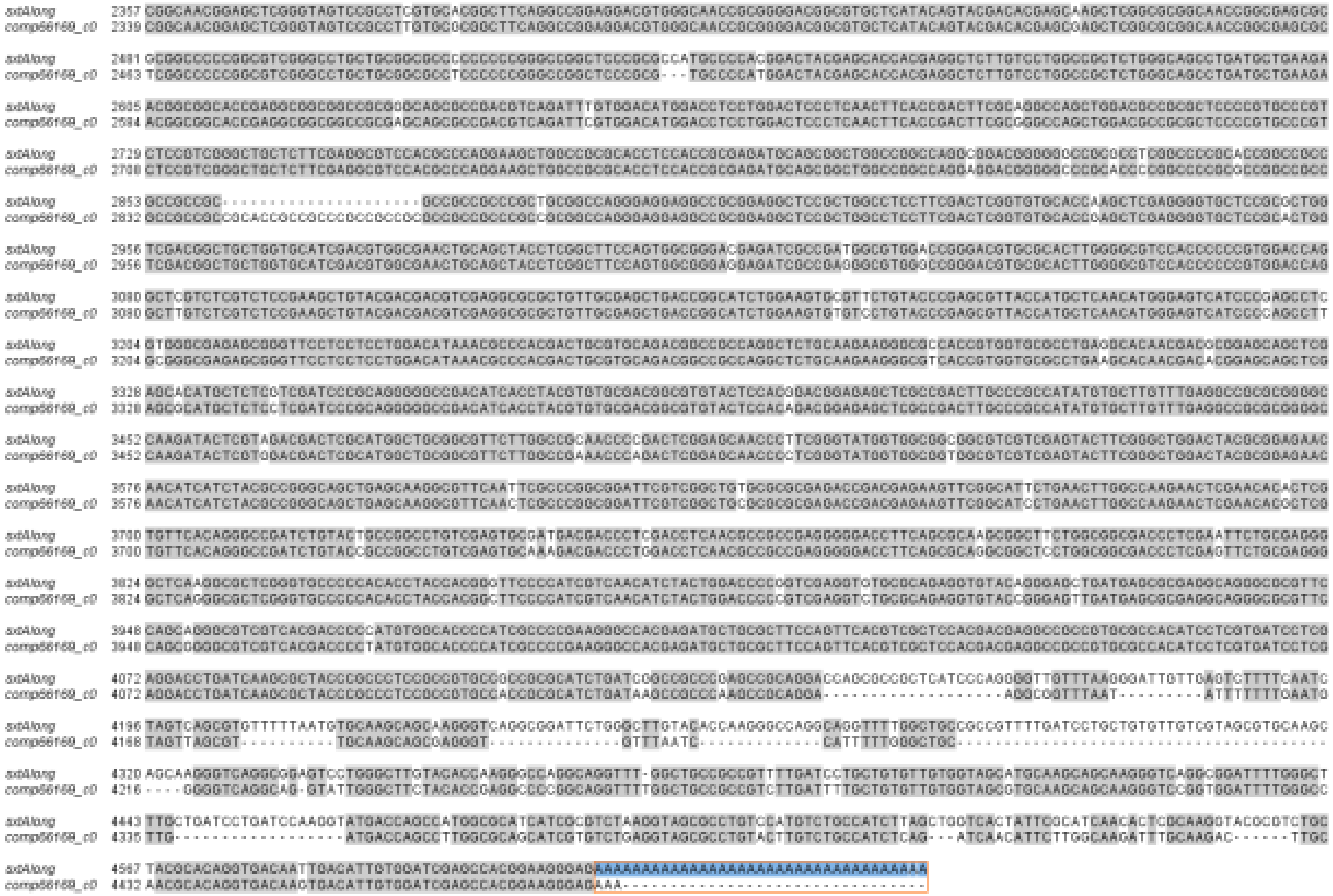

Figure S9. *Cont.*

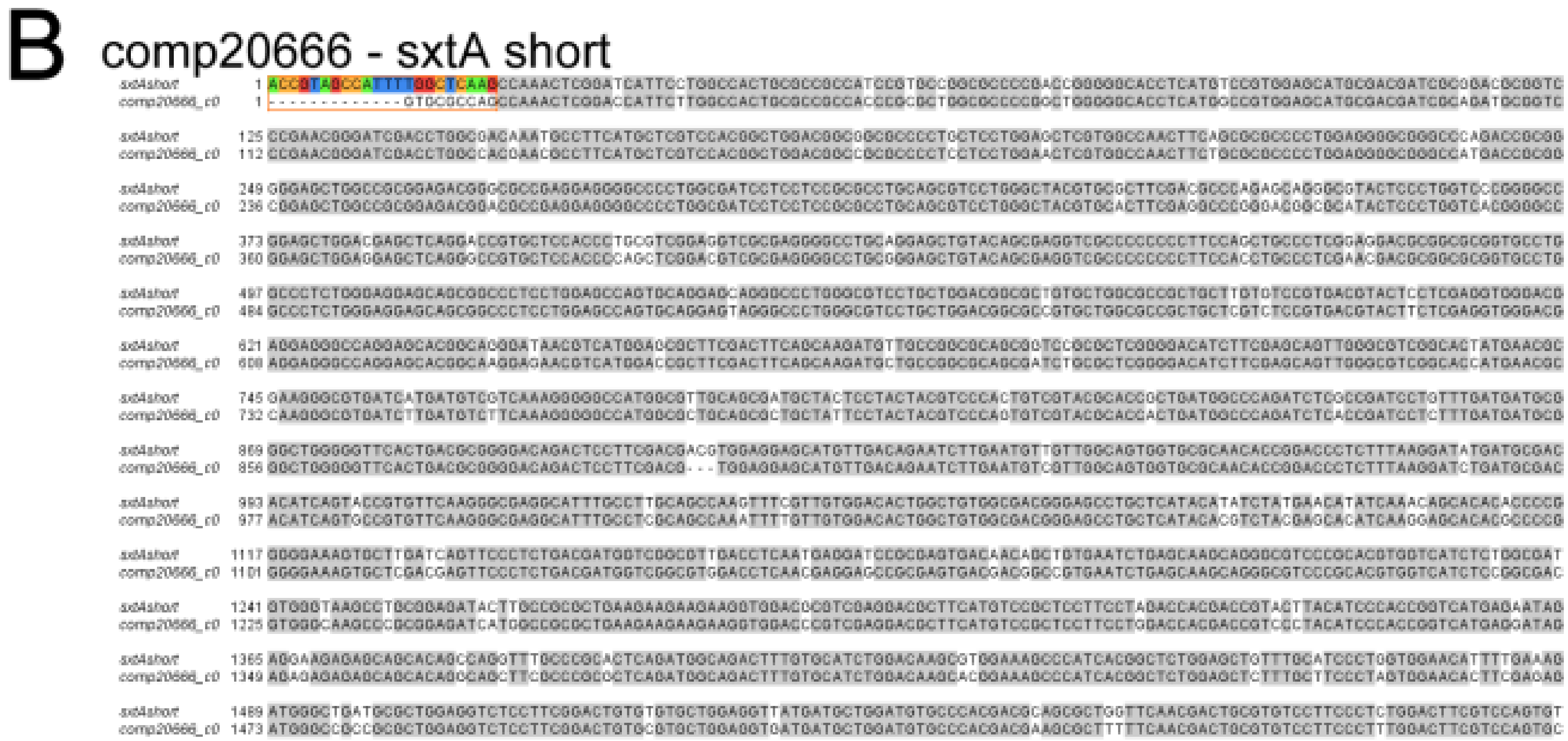

Figure S9. *Cont.*

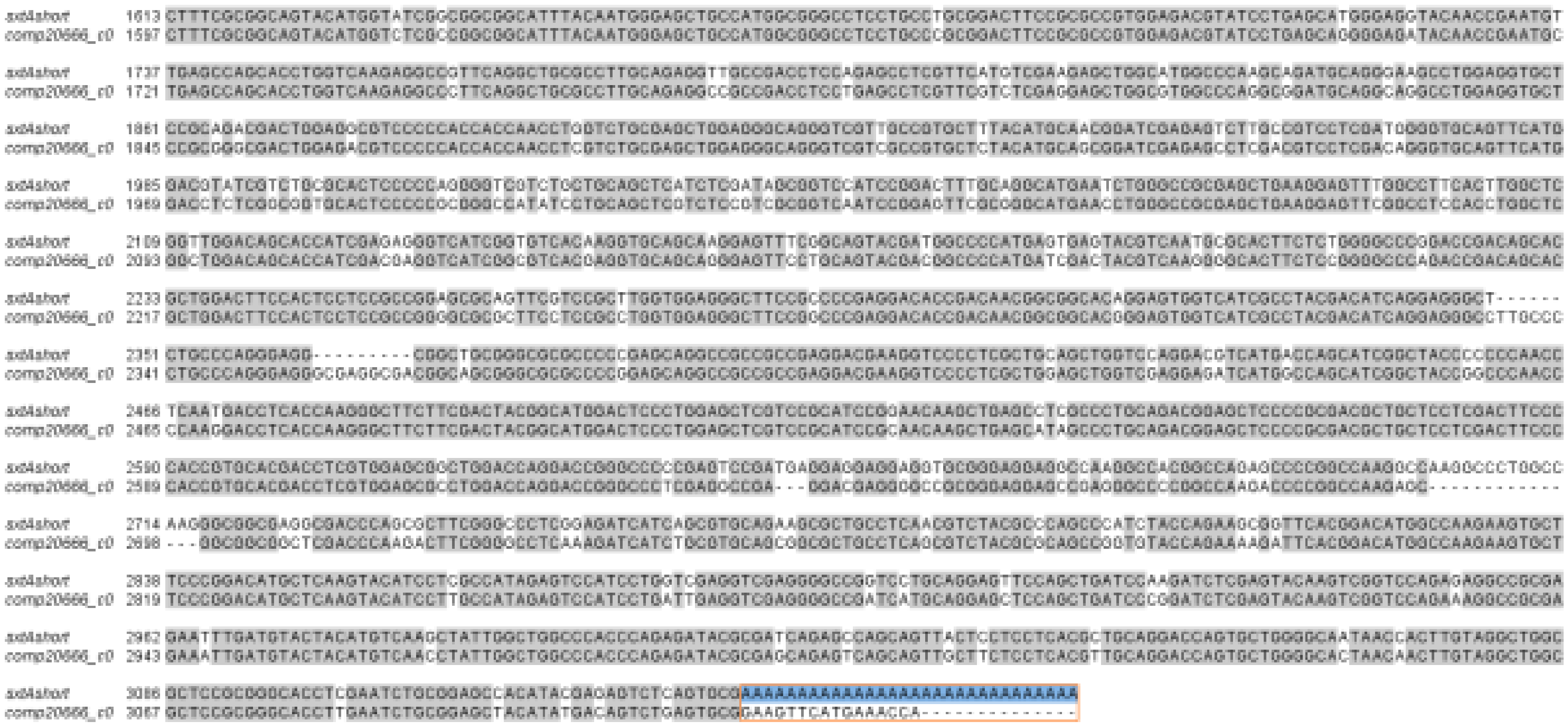

Figure S9. *Cont.*

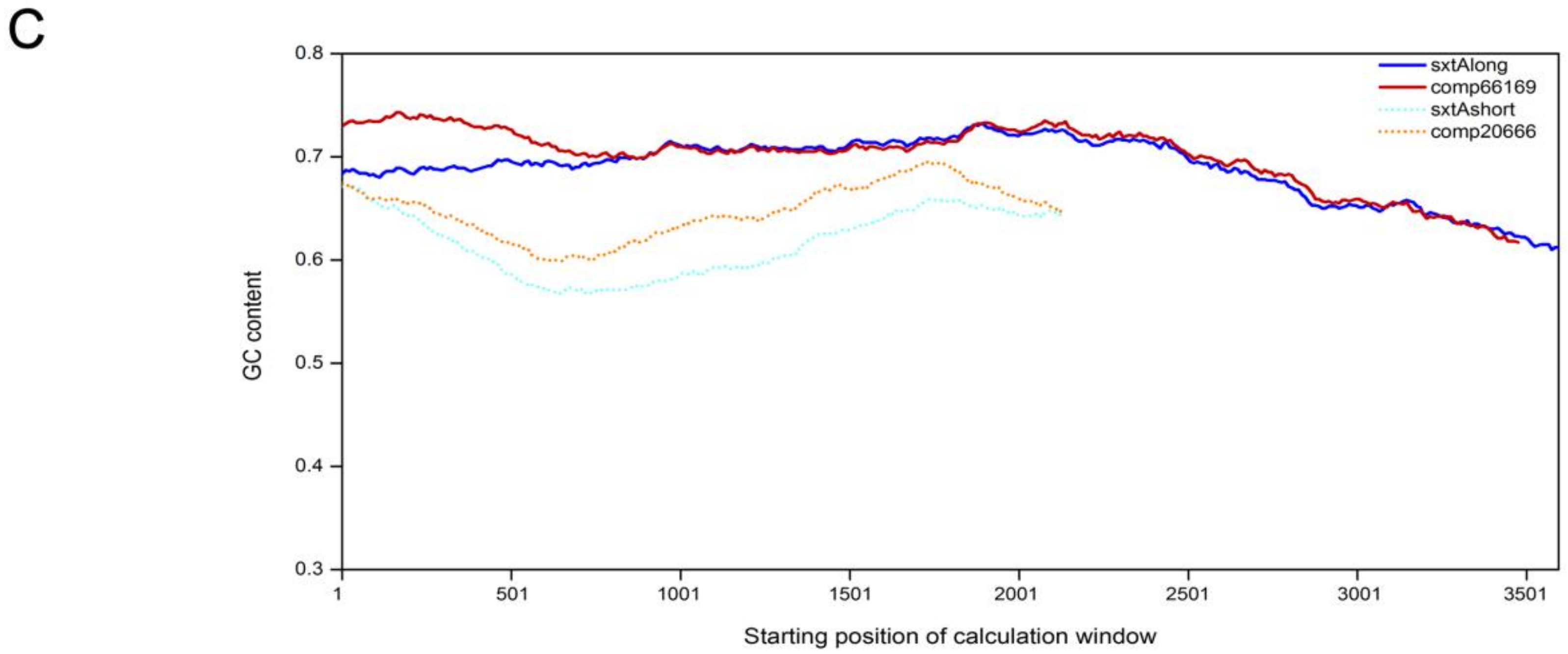

S1. Sequences of *sxtA1* and *sxtA4* motifs in *A. catenella*

>ACHK-NT-sxtA1

TGTACTCCTACTACGTCCCGACGTCCTACGCCCCACTGCTGGACAGGTACCACAGCATCCTCTTCGAGAATCCCGGCTGGGGGTTCGCCGG
TGCCGGCCGGGACTCGCAGGAGCAGGAGGTCCACGTCCACCGGACGCTGAACGTGGTGGGCAGCGGGGCGCAGCACCAGACGCTCTTCA
CGGACCTCGTGCGGCTCATTGACTCGGTCTTCGCGGGCGGGGACTTCGCATCGCAGCCGGCGTTCATCGTGGACACGGGGTGCGGCGACG
GCCGCTTGCTCAGGCGCATCTACGAGCACGTGAAGAGCAACACGCCGCGCGGGAAGGCGCTCGCCGAGCACCCGCTCACGATGGTCGGC
GTCGACTTCAACAAGGACTCTCGGGTGGCGACGGAGCTCAACCTGAGCAGGCACGCGGTCCCGCACCTGGTGCTCTTCGGGGACGTCGGC
AAGCCCGCCGACATCATGGAGACCCTCGGGCGGAATGGGGTGGACCCGAGCAGGTCCCTCCACGTGCGCTCCTTCCTA

>ACHK-T-sxtA1

CTACTCCTACTACGTCCCGACCTCCTACGCCCCACTGCTGGACAGGTACCACCGCATCCTCTTCGAGAATCCCGGCTGGGGGTTCGCCGGC
GCCGGCCGGGACTCGCAGGAGCAGGAGGTCCACGTCCACCGGACGCTGAACGTGGTGGGCAGCGGGGCGCAGCACCAGACGCTCTTCAC
GGATCTCGTGCGGCTCATTGACTCGGTCTTCGCGGGCGGGGACTTCGCGGCGCAGCCGGCGTTCGTCGTGGACACGGGGTGCGGCGACGG
CCGCTTGCTCAGGCGCATCTACGAGCACGTGAAGAGCAACACGCCGCGCGGGAAGGCGCTCGGCGAGCACCCGCTCACGATGGTCGGCG
TCGACTTCAACAAGGACTCTCGGGTGGCCACGGAGCTCAACCTGAGCAGGCACGCGGTCCCGCACCTGGTGCTGTTCGGGGACGTCGGCA
AGCCCGCCGACATCATGGAGATCCTCGGGCGGAGGGGGGTGGACCCGAGCAGGTCCCTCCACGTGCGCTCCTTCCAAACCCCACGACCA

>ACHK-NT-sxtA4

CACCAGAGTCATCCCGAGCCTCGTGGGCGAGAGCGGGTTCCTCCTCCTGTACATAAACGCCCACGACTGCGTGCAGACGGCCGCCAGGCT
CTGCAAGAAGGGCGCCACCGTGGTGCGCCTGAAGCACAACGACACGGAACAGCCCGAGCACATGCTCTCGTCGATCCCGCAGGGGGCCG
ACATCACCTACGTGTGCGACGGCGTGTACTCCACGGACGAAGAGCTCGCCGACTTGCCCGCCATATGTGCTTGTTTGAGGCCGCGCGGGG
CCAAGATACTCGTAGACGACTCGCATGGCTGCGGCGTTCTTGGCCGCAACCCCAACTCGGAGCAACCCCTCGGGTATGGTGGCGGCGGCG
TCATCGAGTACTTCGGGCTGGACTACGCGGAGAACAACATCATCTACGCCGGGCAGCTGAGCAAGGCGTTCAATTCGCCCGGCGGATTCG
TCAGCTGTGCGCGCGAGACCGACGAGAATTTCGGCGTTCTGAACTTGGCCAAGAACTCGAACACACTCGTGTTCACAGGGCCGATCTGTA
CTGCCGGCCTGTCGAGTGCGAAGACGACCTTCGACCTCAACGCCGCCGAGGGGGACCTTCAGCGCAAGCGGCTTCTGGCGGCTACCCTCG
AATTCTGCGAGGGGCTCAAGGCGCTCGGGTGCCCCCACACCTACCACGAGTTCCCCATCGTCAACTCAGCGGGGGACCCCA

>ACHK-T-sxtA4

CCCCGATCATCCCGAGCCTCGTGGGCGAGAGCGGGTTCCTCCTCCTGTACATAAACGCCCACGACTGCGTGCAGACGGCCGCCAGGCTCTG
CAAGAAGGGCGCCACCGTGGTGCGCCTGAAGCACAACGACACGGAACAGCCCGAGCACATGCTCTCGTCGATCCCGCAGGGGGCCGACAT
CACCTACGTGTGCGACGGCGTGTACTCCACGGACGAAGAGCTCGCCGACTTGCCCGCCATATGTGCTTGTTTGAGGCCGCGCGGGGCCAAG
ATACTCGTAGACGACTCGCATGGCTGCGGCGTTCTTGGCCGCAACCCCAACTCGGAGCAACCCCTCGGGTATGGTGGCGGCGGCGTCATC
GAGTACTTCGGGCTGGACTACGCGGAGAACAACATCATCTACGCCGGGCAGCTGAGCAAGGCGTTCAATTCGCCCGGCGGATTCGTCAGC
TGTGCGCGCGAGACCGACGAGAATTTCGGCGTTCTGAACTTGGCCAAGAACTCGAACACACTCGTGTTCACAGGGCCGATCTGTACTGCC
GGCCTGTCGAGTGCGAAGACGACCTTCGACCTCAACGCCGCCGAGGGGGACCTTCAGCGCAAGCGGCTTCTGGCGGCTACCCTCGAATTC
TGCGAGGGGCTCAAGGCGCTCGGGTGCCCCCACACCTACCACGAGTTCCCCATCGTCAACC
